# Prevalence and Characteristics of Canine Parvovirus Type 2 in Henan Province, China

**DOI:** 10.1128/spectrum.01856-22

**Published:** 2022-11-15

**Authors:** Pengfei Fu, Dongchang He, Xuan Cheng, Xinrui Niu, Congrong Wang, Yiqian Fu, Kun Li, Heshui Zhu, Weifei Lu, Jiang Wang, Beibei Chu

**Affiliations:** a College of Veterinary Medicine, Henan Agricultural University, Zhengzhou, Henan Province, China; b Key Laboratory of Animal Biochemistry and Nutrition, Ministry of Agriculture and Rural Affairs of the People’s Republic of China, Zhengzhou, Henan Province, China; c Key Laboratory of Animal Growth and Development, The Education Department of Henan Province, Zhengzhou, Henan Province, China; d International Joint Research Center of National Animal Immunology, Henan Agricultural University, Zhengzhou, Henan Province, China; e Animal Infectious Disease Laboratory, College of Veterinary Medicine, Yangzhou University, Yangzhou, China; f State Key Laboratory of Veterinary Etiological Biology, Lanzhou Veterinary Research Institute, Chinese Academy of Agricultural Sciences, Lanzhou, China; Oklahoma State University, College of Veterinary Medicine

**Keywords:** canine parvovirus, VP2, NS1, phylogenetic analysis, epidemiology, virus evolution

## Abstract

To investigate the epidemic profile and genetic diversity of canine parvovirus type 2 (CPV-2), a total of 111 clinical samples collected from dogs suspected of CPV-2 infection in 10 cities of Henan province of China during 2020 to 2021 were screened by PCR. The results showed a CPV-2-positive rate of 88.29% (98/111). Nearly full-length genomes of 98 CPV-2 strains were sequenced and analyzed. CPV-2c strains (91.84%, 90/98) were significantly higher than that of new CPV-2a strains (8.16%, 8/98) in Henan province without detecting other CPV genotypes, indicating that CPV-2c has become the dominant genotype in Henan province. A phylogenetic analysis of NS1 and VP2 amino acids grouped the strains in this study with Asian strains, which clustered into an identical branch. Based on the CPV-2 VP2 sequences in this study and available in the NCBI database, the adaptation analyses showed that 17 positive selection sites and 10 parallel evolution sites were identified in the VP2 protein of CPV-2, of which three sites (sites 5, 370, and 426) were both under positive selection pressure and parallel evolution. Interestingly, two amino acid mutations (A5G and Q370R) were also observed in the VP2 proteins of 82 CPV-2c strains in this study, which differed from the earlier CPV-2c strain (GU380303) in China. In addition, a unique mutation (I447M) was observed in the VP2 protein of five CPV-2c strains, which was first reported in China. This study provides powerful insight to further our understanding of the epidemic status and evolution of CPV-2 in China.

**IMPORTANCE** CPV-2 was the original virus strain identified in dogs, which cause an acute and lethal disease in dogs. Subsequently, the original CPV-2 was replaced throughout the world by novel antigenic variants (e.g., CPV-2a, CPV-2b, new CPV-2a, new CPV-2b, and CPV-2c). Currently, the epidemiological characteristics of CPV-2 in Henan province of China is still unclear. In our study, a total of 98 nearly full-length genomes of CPV-2 strains were obtained to explore prevalence and genetic evolution of CPV-2 in Henan Province. Moreover, the epidemiological and genetic evolution of CPV-2 in China since its discovery was also investigated. The results of this study will provide valuable information regarding the evolution of CPV-2 strains in China.

## INTRODUCTION

Canine parvovirus type 2 (CPV-2) is a non-enveloped, single-stranded DNA virus belonging to the genus *Protoparvovirus* within the family *Parvoviridae*. CPV-2 has a genome of approximately 5,200 nucleotides (nt) in length and contains two open reading frames (ORFs): ORF1 and ORF2, encoding two nonstructural (NS1 and NS2) and two structural (VP1 and VP2) proteins, respectively, through alternative splicing of the same mRNAs (1–3). VP1 contains the complete sequence of VP2, as well as a 143-residue unique N-terminal sequence (4). The VP2 protein accounts for 90% of the viral capsid and represents the most important protective antigen, since it is closely related to antibody escape, receptor binding, host range, and hemagglutination of the virus (5–9).

The CPV nonstructural protein NS1 is a multifunctional protein with site-specific DNA binding, ATPase, nickase, and helicase activities (10), that is essential for viral replication, DNA packaging, cytotoxicity, and pathogenicity (11–15). However, the role of the nonstructural protein NS2 in viral infection remains unclear. A recent study showed that CPV NS2 can interact with chromatin regulating cellular proteins (10).

CPV-2 was the original virus strain identified in dogs, and spread worldwide in the 1970s (16), causing an acute and lethal disease in dogs characterized by vomiting, enteritis, and acute lymphopenia (3). Subsequently, the original CPV-2 was replaced throughout the world by novel antigenic variants of CPV-2a based on VP2 amino acid (aa) substitutions (Met-87-Leu, Ile-101-Thr, Ala-300-Gly, Asp-305-Tyr, and Asn-375-Asp) (17–22). Following the discovery of CPV-2a, two other antigenic variants, termed CPV-2b (VP2 Asn-426-Asp) and CPV-2c (VP2 Asp-426-Glu), were identified. Among them, CPV-2b was first detected in 1984 in the United States (18) and CPV-2c was identified in 2000 in Italy (23). Compared to the original CPV-2, the antigenic mutants CPV-2a, CPV-2b, and CPV-2c are more pathogenic to dogs and have an extended host range that includes cats (24–26). After the spread of CPV-2c, variants of new CPV-2a and new CPV-2b emerged (27), which differed from CPV-2a and CPV-2b by a single amino acid (VP2 Ser-297-Ala) (28–30).

In China, CPV-2 was first described in 1982 (31). In 1986, CPV-2a replaced CPV-2 as the predominant genotype (31), followed by the emergence of CPV-2b emerged in 1997 and circulated together with CPV-2a (32). CPV-2c was subsequently identified in Jilin province, China (33). In 2015, new CPV-2a and new CPV-2b were discovered in Gansu province (34). Previous studies have suggested that CPV-2c was identified as the dominant genotype in Europe and the Americas (35–37), whereas CPV-2a/new CPV-2a remained the predominant genotypes in China (20, 38–40). In addition, several recent studies have reported that the prevalence of CPV-2c has increased within different regions of China (41–43). To date, the genomic characteristics of CPV-2 in Henan province of China remain unclear. As a result, nearly full-length genomes of 98 CPV-2 strains from 10 cities in Henan province between 2020 and 2021 were sequenced in our study. Combined with the CPV-2 NS1 and VP2 gene sequences available from the NCBI database, a detailed study was conducted on the molecular evolution, population dynamics, positive selection, and parallel mutations of the amino acid sites of CPV-2 in China.

## RESULTS

### High prevalence of the CPV-2c in Henan province, China.

Of the 111 samples with suspected CPV-2 infection, 98 were positive for CPV-2 with a prevalence rate of 88.29%. The results of enterovirus detection in dogs revealed that most of the dogs (*n* = 77) were infected with CPV alone. Within the 98 CPV-2-positive samples, the positive rates for canine adenovirus 1 and 2 (CAV-1/2) and canine coronavirus (CoV) were 6.12% (6/98) and 12.24% (12/98), respectively. Furthermore, three dogs were coinfected with CPV-2, CAV-1/2, and CoV. Canine rotavirus (CRV) was not detected in any of the collected samples (see Table S1 in the supplemental material).

Near full-length genome sequences (4,269 or 4,270 nt) of 98 CPV-2 strains acquired in this study (accession numbers ON322751 to ON322848) have been summarized in Table 1. The alignment analyses displayed that 98 CPV-2 strains had 98.9 to 100% genomic nucleotide identity with each other, and contained four putative ORFs, which encoded NS1, NS2, VP1, and VP2. Analysis of the deduced amino acid sequences of the VP2 gene revealed that 91.84% (90/98) of the CPV-2 stains with 87-Leu, 101-Thr, 300-Gly, 305-Tyr, 375-Asp, and 426-Glu residues in the VP2 proteins belonged to the CPV-2c genotype. In contrast, 8.16% strains (8/98) with 87-Leu, 101-Thr, 297-Ala, 300-Gly, 305-Tyr, 375-Asp, and 426-Asn residues belonged to the new CPV-2a genotype. No other CPV-2 genotypes were detected in this study.

**TABLE 1:** Amino acid mutations of the VP2 gene detected in this study^*a*^

Strain	Genetic type	GenBank accession no.	Yr	Origin	Amino acid position of VP2															
					4	5	13	87	101	267	297	300	305	324	370	375	426	440	447	555
Reference																				
CPV-b	Original CPV2	M38245	1978	USA	G	A	P	M	I	F	S	A	D	Y	Q	N	N	T	I	V
CPV-15	CPV 2a	M24003	1984	USA	G	A	P	L	T	F	S	G	Y	Y	Q	D	N	T	I	I
BR6-80	CPV 2a	DQ340404	1980	Brazil	G	A	P	L	T	F	S	G	Y	Y	Q	D	N	T	I	V
CPV-39	CPV 2b	M74849	1984	USA	G	A	P	L	T	F	S	G	Y	Y	Q	D	D	T	I	V
BR8-90	New CPV 2a	DQ340411	1990	Brazil	G	A	P	L	T	F	A	G	Y	Y	Q	D	N	T	I	V
BJ03/17	New CPV 2a	MF134808	2017	China	G	A	P	L	T	Y	A	G	Y	I	Q	D	N	A	I	V
LZ2	New CPV 2b	JQ268284	2011	China	G	A	P	L	T	Y	A	G	Y	I	Q	D	D	A	I	V
288-01	CPV 2c	MF177239	2001	Italy	G	A	P	L	T	F	A	G	Y	Y	Q	D	E	T	I	V
06/09	CPV 2c	GU380303	2009	China	G	A	P	L	T	Y	A	G	Y	I	Q	D	E	T	I	V
Canine/China/23	CPV 2c	MH476592	2017	China	G	G	P	L	T	Y	A	G	Y	I	R	D	E	T	I	V
HCM01	CPV 2c	MT106234	2017	Vietnam	G	G	P	L	T	Y	A	G	Y	I	R	D	E	T	M	V
This study																				
HN-002	CPV 2c	ON322751	2020/June	China	G	G	P	L	T	Y	A	G	Y	I	R	D	E	T	I	V
HN-003	New CPV 2a	ON322752	2020/June	China	G	A	P	L	T	Y	A	G	Y	I	Q	D	N	A	I	V
HN-004	CPV 2c	ON322753	2020/June	China	G	G	P	L	T	Y	A	G	Y	I	R	D	E	T	I	V
HN-005	CPV 2c	ON322754	2020/June	China	G	G	P	L	T	Y	A	G	Y	I	R	D	E	T	I	V
HN-006	CPV 2c	ON322755	2020/June	China	G	G	P	L	T	Y	A	G	Y	I	R	D	E	T	I	V
HN-007	CPV 2c	ON322756	2020/June	China	G	G	P	L	T	Y	A	G	Y	I	R	D	E	T	I	V
HN-008	CPV 2c	ON322757	2020/June	China	G	G	P	L	T	Y	A	G	Y	I	R	D	E	T	I	V
HN-009	CPV 2c	ON322758	2020/July	China	G	G	P	L	T	Y	A	G	Y	I	R	D	E	T	I	V
HN-010	CPV 2c	ON322759	2020/July	China	G	G	P	L	T	Y	A	G	Y	I	R	D	E	T	I	V
HN-011	CPV 2c	ON322760	2020/July	China	G	G	P	L	T	Y	A	G	Y	I	R	D	E	T	I	V
HN-012	CPV 2c	ON322761	2020/Aug	China	G	G	P	L	T	Y	A	G	Y	I	R	D	E	T	I	V
HN-013	New CPV 2a	ON322762	2020/Aug	China	G	A	P	L	T	Y	A	G	Y	I	Q	D	N	A	I	V
HN-014	New CPV 2a	ON322763	2020/Aug	China	G	A	P	L	T	Y	A	G	Y	I	Q	D	N	A	I	V
HN-015	New CPV 2a	ON322764	2020/Aug	China	G	A	P	L	T	Y	A	G	Y	I	Q	D	N	A	I	V
HN-016	New CPV 2a	ON322765	2020/Aug	China	G	A	P	L	T	Y	A	G	Y	I	Q	D	N	A	I	V
HN-017	CPV 2c	ON322766	2020/July	China	G	G	P	L	T	Y	A	G	Y	I	R	D	E	T	I	V
HN-018	CPV 2c	ON322767	2020/Aug	China	G	G	P	L	T	Y	A	G	Y	I	R	D	E	T	I	V
HN-019	CPV 2c	ON322768	2020/Aug	China	G	A	P	L	T	Y	A	G	Y	I	R	D	E	T	M	V
HN-021	CPV 2c	ON322769	2020/Aug	China	G	G	P	L	T	Y	A	G	Y	I	R	D	E	T	I	V
HN-022	CPV 2c	ON322770	2020/Aug	China	G	G	P	L	T	Y	A	G	Y	I	R	D	E	T	I	V
HN-023	CPV 2c	ON322771	2020/Aug	China	G	G	P	L	T	Y	A	G	Y	I	R	D	E	T	I	V
HN-024	CPV 2c	ON322772	2020/Aug	China	G	G	P	L	T	Y	A	G	Y	I	R	D	E	T	I	V
HN-025	CPV 2c	ON322773	2020/Aug	China	G	G	P	L	T	Y	A	G	Y	I	R	D	E	T	I	V
HN-026	CPV 2c	ON322774	2020/July	China	G	G	P	L	T	Y	A	G	Y	I	R	D	E	T	I	V
HN-027	New CPV 2a	ON322775	2020/July	China	G	A	P	L	T	Y	A	G	Y	I	Q	D	N	A	I	V
HN-028	CPV 2c	ON322776	2020/Aug	China	G	G	P	L	T	Y	A	G	Y	I	R	D	E	T	I	V
HN-029	CPV 2c	ON322777	2020/Aug	China	G	G	P	L	T	Y	A	G	Y	I	R	D	E	T	I	V
HN-030	CPV 2c	ON322778	2020/Aug	China	G	G	P	L	T	Y	A	G	Y	I	R	D	E	T	I	V
HN-031	CPV 2c	ON322779	2020/Aug	China	G	G	P	L	T	Y	A	G	Y	I	R	D	E	T	I	V
HN-033	CPV 2c	ON322780	2020/Aug	China	G	G	P	L	T	Y	A	G	Y	I	R	D	E	T	I	V
HN-034	CPV 2c	ON322781	2020/Aug	China	G	G	P	L	T	Y	A	G	Y	I	R	D	E	T	I	V
HN-035	CPV 2c	ON322782	2020/July	China	G	G	P	L	T	Y	A	G	Y	I	R	D	E	T	I	V
HN-036	New CPV 2a	ON322783	2020/Aug	China	G	A	P	L	T	Y	A	G	Y	I	Q	D	N	A	I	V
HN-038	CPV 2c	ON322784	2020/Aug	China	G	G	P	L	T	Y	A	G	Y	I	R	D	E	T	I	V
HN-039	CPV 2c	ON322785	2020/Aug	China	G	G	P	L	T	Y	A	G	Y	I	R	D	E	T	I	V
HN-040	CPV 2c	ON322786	2020/Aug	China	G	G	P	L	T	Y	A	G	Y	I	R	D	E	T	I	V
HN-041	CPV 2c	ON322787	2020/Aug	China	G	G	P	L	T	Y	A	G	Y	I	R	D	E	T	I	V
HN-042	CPV 2c	ON322788	2020/Aug	China	G	G	P	L	T	Y	A	G	Y	I	R	D	E	T	I	V
HN-043	CPV 2c	ON322789	2020/Aug	China	G	G	P	L	T	Y	A	G	Y	I	R	D	E	T	I	V
HN-044	CPV 2c	ON322790	2020/Aug	China	G	G	P	L	T	Y	A	G	Y	I	R	D	E	T	I	V
HN-045	CPV 2c	ON322791	2020/Aug	China	G	G	P	L	T	Y	A	G	Y	I	R	D	E	T	I	V
HN-046	CPV 2c	ON322792	2020/Aug	China	G	G	P	L	T	Y	A	G	Y	I	R	D	E	T	I	V
HN-047	CPV 2c	ON322793	2020/Aug	China	G	G	P	L	T	Y	A	G	Y	I	R	D	E	T	I	V
HN-048	CPV 2c	ON322794	2020/Aug	China	G	G	P	L	T	Y	A	G	Y	I	R	D	E	T	I	V
HN-049	CPV 2c	ON322795	2020/Sep	China	G	G	S	L	T	Y	A	G	Y	I	R	D	E	T	I	V
HN-050	CPV 2c	ON322796	2020/Sep	China	G	G	P	L	T	Y	A	G	Y	I	R	D	E	T	I	V
HN-051	CPV 2c	ON322797	2020/Sep	China	G	G	P	L	T	Y	A	G	Y	I	R	D	E	T	M	V
HN-055	CPV 2c	ON322798	2020/Sep	China	G	G	P	L	T	Y	A	G	Y	I	R	D	E	T	I	V
HN-056	CPV 2c	ON322799	2020/Oct	China	G	A	P	L	T	Y	A	G	Y	I	R	D	E	T	M	V
HN-057	CPV 2c	ON322800	2020/Oct	China	G	A	P	L	T	Y	A	G	Y	I	R	D	E	T	M	V
HN-058	CPV 2c	ON322801	2020/Oct	China	G	A	P	L	T	Y	A	G	Y	I	R	D	E	T	M	V
HN-059	CPV 2c	ON322802	2020/Oct	China	G	G	P	L	T	Y	A	G	Y	I	R	D	E	T	I	V
HN-060	CPV 2c	ON322803	2020/Oct	China	G	G	P	L	T	Y	A	G	Y	I	R	D	E	T	I	V
HN-061	CPV 2c	ON322804	2020/Oct	China	G	G	P	L	T	Y	A	G	Y	I	R	D	E	T	I	V
HN-062	CPV 2c	ON322805	2020/Oct	China	G	G	P	L	T	Y	A	G	Y	I	R	D	E	T	I	V
HN-063	CPV 2c	ON322806	2020/Oct	China	G	G	P	L	T	Y	A	G	Y	I	R	D	E	T	I	V
HN-064	CPV 2c	ON322807	2020/Oct	China	G	G	P	L	T	Y	A	G	Y	I	R	D	E	T	I	V
HN-065	CPV 2c	ON322808	2020/Oct	China	G	G	P	L	T	Y	A	G	Y	I	R	D	E	T	I	V
HN-066	CPV 2c	ON322809	2020/Oct	China	G	G	P	L	T	Y	A	G	Y	I	R	D	E	T	I	V
HN-068	CPV 2c	ON322810	2020/Aug	China	G	G	P	L	T	Y	A	G	Y	I	R	D	E	T	I	V
HN-069	CPV 2c	ON322811	2020/Sep	China	G	G	P	L	T	Y	A	G	Y	I	R	D	E	T	I	V
HN-070	CPV 2c	ON322812	2020/Aug	China	G	G	P	L	T	Y	A	G	Y	I	R	D	E	T	I	V
HN-071	CPV 2c	ON322813	2020/Sep	China	G	G	P	L	T	Y	A	G	Y	I	R	D	E	T	I	V
HN-072	CPV 2c	ON322814	2020/Sep	China	G	G	P	L	T	Y	A	G	Y	I	R	D	E	T	I	V
HN-073	CPV 2c	ON322815	2020/Sep	China	G	G	P	L	T	Y	A	G	Y	I	R	D	E	T	I	V
HN-074	CPV 2c	ON322816	2020/Sep	China	G	G	P	L	T	Y	A	G	Y	I	R	D	E	T	I	V
HN-075	CPV 2c	ON322817	2020/Sep	China	G	G	P	L	T	Y	A	G	Y	I	R	D	E	T	I	V
HN-076	CPV 2c	ON322818	2020/Sep	China	G	G	P	L	T	Y	A	G	Y	I	R	D	E	T	I	V
HN-077	CPV 2c	ON322819	2020/Oct	China	G	G	P	L	T	Y	A	G	Y	I	R	D	E	T	I	V
HN-078	CPV 2c	ON322820	2020/Aug	China	G	G	P	L	T	Y	A	G	Y	I	R	D	E	T	I	V
HN-079	CPV 2c	ON322821	2020/Nov	China	G	G	P	L	T	Y	A	G	Y	I	R	D	E	T	I	V
HN-080	CPV 2c	ON322822	2020/Nov	China	G	G	P	L	T	Y	A	G	Y	I	R	D	E	T	I	V
HN-081	CPV 2c	ON322823	2020/Nov	China	G	G	P	L	T	Y	A	G	Y	I	R	D	E	T	I	V
HN-082	CPV 2c	ON322824	2020/Nov	China	G	G	P	L	T	Y	A	G	Y	I	R	D	E	T	I	V
HN-084	CPV 2c	ON322825	2020/Nov	China	E	G	P	L	T	Y	A	G	Y	I	R	D	E	T	I	V
HN-085	CPV 2c	ON322826	2020/Nov	China	G	G	P	L	T	Y	A	G	Y	I	R	D	E	T	I	V
HN-086	CPV 2c	ON322827	2020/Nov	China	G	A	P	L	T	Y	A	G	Y	I	R	D	E	T	I	V
HN-087	CPV 2c	ON322828	2020/Nov	China	G	G	P	L	T	Y	A	G	Y	I	R	D	E	T	I	V
HN-088	CPV 2c	ON322829	2020/Nov	China	G	G	P	L	T	Y	A	G	Y	I	R	D	E	T	I	V
HN-089	New CPV 2a	ON322830	2020/Nov	China	G	A	P	L	T	Y	A	G	Y	I	Q	D	N	A	I	V
HN-090	CPV 2c	ON322831	2020/Nov	China	G	G	P	L	T	Y	A	G	Y	I	R	D	E	T	I	V
HN-091	CPV 2c	ON322832	2020/Nov	China	G	G	P	L	T	Y	A	G	Y	I	R	D	E	T	I	V
HN-092	CPV 2c	ON322833	2020/Nov	China	G	G	P	L	T	Y	A	G	Y	I	R	D	E	T	I	V
HN-093	CPV 2c	ON322834	2020/Nov	China	G	G	P	L	T	Y	A	G	Y	I	R	D	E	T	I	V
HN-097	CPV 2c	ON322835	2020/Oct	China	G	G	P	L	T	Y	A	G	Y	I	R	D	E	T	I	V
HN-098	CPV 2c	ON322836	2020/Nov	China	G	G	P	L	T	Y	A	G	Y	I	R	D	E	T	I	V
HN-099	CPV 2c	ON322837	2021/Jan	China	G	G	P	L	T	Y	A	G	Y	I	R	D	E	T	I	V
HN-100	CPV 2c	ON322838	2021/Jan	China	G	G	P	L	T	Y	A	G	Y	I	R	D	E	T	I	V
HN-102	CPV 2c	ON322839	2021/Jan	China	G	G	P	L	T	Y	A	G	Y	I	R	D	E	T	I	V
HN-103	CPV 2c	ON322840	2021/Jan	China	G	G	P	L	T	Y	A	G	Y	I	R	D	E	T	I	V
HN-104	CPV 2c	ON322841	2021/Jan	China	G	G	P	L	T	Y	A	G	Y	I	R	D	E	T	I	V
HN-105	CPV 2c	ON322842	2021/Jan	China	G	G	P	L	T	Y	A	G	Y	I	R	D	E	T	I	V
HN-106	CPV 2c	ON322843	2021/Jan	China	G	G	P	L	T	Y	A	G	Y	I	R	D	E	T	I	V
HN-107	CPV 2c	ON322844	2021/Jan	China	G	G	P	L	T	Y	A	G	Y	I	R	D	E	T	I	V
HN-108	CPV 2c	ON322845	2021/Jan	China	G	G	P	L	T	Y	A	G	Y	I	R	D	E	T	I	V
HN-109	CPV 2c	ON322846	2021/Jan	China	G	G	P	L	T	Y	A	G	Y	I	R	D	E	T	I	V
HN-110	CPV 2c	ON322847	2020/Aug	China	G	G	P	L	T	Y	A	G	Y	I	R	D	E	T	I	V
HN-111	CPV 2c	ON322848	2020/Nov	China	G	G	P	L	T	Y	A	G	Y	I	R	D	E	T	I	V

a Shading indicates a mutation compared to the original CPV-2 (GenBank accession number M38245).

### Sequence comparison and amino acid sequence analysis of VP2.

The eight new CPV-2a strains generated in this study shared 99.9 to 100.0% nucleotide and 100.0% amino acid identity with each other and had a 100% amino acid sequence identity with the new CPV-2a reference strains (2008-KY403998-China, 2010-KM457102-Uruguay, 2013-LC214970-Vietnam, 2018-MH545963-India, and 2018-MK895485-Nigeria). Compared to the reference CPV-2 strain (original CPV2, GenBank no. M38245), except for the characteristic VP2 amino acid mutations of new CPV-2a (M87L, I101T, S297A, A300G, D305Y, and N375D), three mutations F267Y, Y324I, and T440A were found in the VP2 protein of all the new CPV-2a strains (*n* = 8) in this study (Table 1).

VP2 proteins of the CPV-2c strains derived from Europe and Americas primarily differed from those in Asia by three mutations (F267Y, Y324I, and Q370R) (7, 44). Based on these mutations, all of the 90 CPV-2c strains belonged to the Asian CPV-2c genotype. The 90 CPV-2c strains shared 99.4 to 100.0% nucleotide homology and 99.5 to 100.0% amino acid homology. Five of the CPV-2c strains (HN-019, HN-056, CHN-057, HN-058, and HN-086) acquired in this study contained the traditional amino acid (A) at position 5 of VP2, which was consistent with the original CPV-2 (M38245), CPV-2c G7/97 (FJ005196), and CPV-2 vaccines (FJ197846). However, 94.44% (85/90) of the 90 CPV-2c strains contained an amino acid mutation (A5G). No amino acid change (A5G) was observed in the new CPV-2a strain.

Notably, an amino acid analysis of the VP2 protein revealed that I447M was observed in five of the CPV-2c strains (HN-019, HN-051, HN-056, HN-057, and HN-058), P13S in one CPV-2c strain (HN-049), and G4E in one CPV-2c strain (HN-084). Compared to the earlier CPV-2c strains circulating in China, 82 CPV-2 strains in this study contained two amino acid mutations (A5G and Q370R) (Table 1).

### Specific amino acid mutations in the VP1 proteins.

The amino acid residue mutations in VP1 were shown in Table 2. Two mutations (A131T and A131N) appeared in the 131^st^ amino acid residue of the VP1 protein, of which the A131T mutation existed in 80 CPV-2c strains, while the A131N mutation was identified in two CPV-2c strains (HN039 and HN070). Compared to the original CPV2 (M38245), in this study, three new CPV 2a (HN003, HN036, and HN089) and CPV 2c (HN038, HN070, and HN086) strains were found to have the K116R mutation in the VP1 protein. The same mutations were also identified in the LZ2 reference strain (new CPV 2b, JQ268284). Two uncommon NS1 A88T and R138K mutations were detected in new CPV-2a (HN089) and CPV-2c (HN035) strains, respectively. It has previously been reported that Vietnam CPV-2c strains with a I447M mutation in VP2 protein have a T112I mutation in their VP1 protein; however, no VP1 T112I mutation was detected in the CPV 2c strains with a VP2 I447M mutation in this study. Moreover, some aa changes (G147E, A148G, P156S, M230L, I244T, F410Y, S440A, A443G, D448Y, Y467I, Q513R, N518D, N569E, T583A, and I590M) of the VP1 protein were lying in the corresponding encoded VP2 protein (G4E, A5G, P13S, M87L, I101T, F267Y, S297A, A300G, D305Y, Y324I, Q370R, N375D, N426E, T440A, and I447M).

**TABLE 2:** Amino acid mutations of the VP1 gene detected in this study^*a*^

Strain	Genetic type	GenBank accession no.	Yr	Origin	Amino acid position of VP1									
					20	26	50	52	88	112	116	125	131	138
Reference														
CPV-b	Original CPV2	M38245	1978	USA	L	L	L	S	A	T	K	L	A	R
CPV-15	CPV 2a	M24003	1984	USA	L	L	L	S	A	T	K	L	A	R
BR6-80	CPV 2a	DQ340404	1980	Brazil	L	L	L	S	A	T	K	L	A	R
CPV-39	CPV 2b	M74849	1984	USA	L	L	L	S	A	T	K	L	A	R
BR8-90	New CPV 2a	DQ340411	1990	Brazil	L	L	L	S	A	T	K	L	A	R
BJ03/17	New CPV 2a	MF134808	2017	China	L	L	L	S	A	T	K	L	A	R
LZ2	New CPV 2b	JQ268284	2011	China	L	L	L	S	A	T	R	L	A	R
288-01	CPV 2c	MF177239	2001	Italy	L	L	L	S	A	T	K	L	A	R
Canine/China/23	CPV 2c	MH476592	2017	China	L	L	L	S	A	T	K	L	T	R
HCM01	CPV 2c	MT106234	2017	Vietnam	L	L	L	S	A	I	K	F	T	R
This study														
HN-002	CPV 2c	ON322751	2020/June	China	L	L	L	S	A	T	K	L	T	R
HN-003	New CPV 2a	ON322752	2020/June	China	L	L	L	S	A	T	R	L	A	R
HN-004	CPV 2c	ON322753	2020/June	China	L	L	L	S	A	T	K	L	T	R
HN-005	CPV 2c	ON322754	2020/June	China	L	L	L	S	A	T	K	L	T	R
HN-006	CPV 2c	ON322755	2020/June	China	L	L	L	S	A	T	K	L	T	R
HN-007	CPV 2c	ON322756	2020/June	China	L	L	L	S	A	T	K	L	T	R
HN-008	CPV 2c	ON322757	2020/June	China	L	L	L	S	A	T	K	L	T	R
HN-009	CPV 2c	ON322758	2020/July	China	L	L	L	S	A	T	K	L	T	R
HN-010	CPV 2c	ON322759	2020/July	China	L	L	L	S	A	T	K	L	T	R
HN-011	CPV 2c	ON322760	2020/July	China	L	L	L	S	A	T	K	L	T	R
HN-012	CPV 2c	ON322761	2020/Aug	China	L	L	L	S	A	T	K	L	T	R
HN-013	New CPV 2a	ON322762	2020/Aug	China	L	L	L	S	A	T	K	L	A	R
HN-014	New CPV 2a	ON322763	2020/Aug	China	L	L	L	S	A	T	K	L	A	R
HN-015	New CPV 2a	ON322764	2020/Aug	China	L	L	L	S	A	T	K	L	A	R
HN-016	New CPV 2a	ON322765	2020/Aug	China	L	L	L	S	A	T	K	L	A	R
HN-017	CPV 2c	ON322766	2020/July	China	L	L	L	S	A	T	K	L	T	R
HN-018	CPV 2c	ON322767	2020/Aug	China	L	L	L	S	A	T	K	L	T	R
HN-019	CPV 2c	ON322768	2020/Aug	China	L	L	L	S	A	T	K	L	A	R
HN-021	CPV 2c	ON322769	2020/Aug	China	L	L	L	S	A	T	K	L	T	R
HN-022	CPV 2c	ON322770	2020/Aug	China	L	L	L	S	A	T	K	L	T	R
HN-023	CPV 2c	ON322771	2020/Aug	China	L	L	L	S	A	T	K	L	T	R
HN-024	CPV 2c	ON322772	2020/Aug	China	L	L	L	S	A	T	K	L	T	R
HN-025	CPV 2c	ON322773	2020/Aug	China	L	L	L	S	A	T	K	L	T	R
HN-026	CPV 2c	ON322774	2020/July	China	L	L	L	S	A	T	K	L	T	R
HN-027	New CPV 2a	ON322775	2020/July	China	L	L	L	S	A	T	K	L	A	R
HN-028	CPV 2c	ON322776	2020/Aug	China	L	L	L	S	A	T	K	L	T	R
HN-029	CPV 2c	ON322777	2020/Aug	China	F	F	F	F	A	T	K	L	T	R
HN-030	CPV 2c	ON322778	2020/Aug	China	L	L	L	S	A	T	K	L	T	R
HN-031	CPV 2c	ON322779	2020/Aug	China	L	L	L	S	A	T	K	L	T	R
HN-033	CPV 2c	ON322780	2020/Aug	China	L	L	L	S	A	T	K	L	T	R
HN-034	CPV 2c	ON322781	2020/Aug	China	L	L	L	S	A	T	K	L	T	R
HN-035	CPV 2c	ON322782	2020/July	China	L	L	L	S	A	T	K	L	T	K
HN-036	New CPV 2a	ON322783	2020/Aug	China	L	L	L	S	A	T	R	L	A	R
HN-038	CPV 2c	ON322784	2020/Aug	China	L	L	L	S	A	T	R	L	A	R
HN-039	CPV 2c	ON322785	2020/Aug	China	L	L	L	S	A	T	K	L	N	R
HN-040	CPV 2c	ON322786	2020/Aug	China	L	L	L	S	A	T	K	L	A	R
HN-041	CPV 2c	ON322787	2020/Aug	China	L	L	L	S	A	T	K	L	T	R
HN-042	CPV 2c	ON322788	2020/Aug	China	L	L	L	S	A	T	K	L	T	R
HN-043	CPV 2c	ON322789	2020/Aug	China	L	L	L	S	A	T	K	L	T	R
HN-044	CPV 2c	ON322790	2020/Aug	China	L	L	L	S	A	T	K	L	T	R
HN-045	CPV 2c	ON322791	2020/Aug	China	L	L	L	S	A	T	K	L	T	R
HN-046	CPV 2c	ON322792	2020/Aug	China	L	L	L	S	A	T	K	L	T	R
HN-047	CPV 2c	ON322793	2020/Aug	China	L	L	L	S	A	T	K	L	T	R
HN-048	CPV 2c	ON322794	2020/Aug	China	L	L	L	S	A	T	K	L	T	R
HN-049	CPV 2c	ON322795	2020/Sep	China	L	L	L	S	A	T	K	L	T	R
HN-050	CPV 2c	ON322796	2020/Sep	China	L	L	L	S	A	T	K	L	T	R
HN-051	CPV 2c	ON322797	2020/Sep	China	L	L	L	S	A	T	K	L	T	R
HN-055	CPV 2c	ON322798	2020/Sep	China	L	L	L	S	A	T	K	L	T	R
HN-056	CPV 2c	ON322799	2020/Oct	China	L	L	L	S	A	T	K	L	A	R
HN-057	CPV 2c	ON322800	2020/Oct	China	L	L	L	S	A	T	K	L	A	R
HN-058	CPV 2c	ON322801	2020/Oct	China	L	L	L	S	A	T	K	L	A	R
HN-059	CPV 2c	ON322802	2020/Oct	China	L	L	L	S	A	T	K	L	T	R
HN-060	CPV 2c	ON322803	2020/Oct	China	L	L	L	S	A	T	K	L	T	R
HN-061	CPV 2c	ON322804	2020/Oct	China	L	L	L	S	A	T	K	L	T	R
HN-062	CPV 2c	ON322805	2020/Oct	China	L	L	L	S	A	T	K	L	T	R
HN-063	CPV 2c	ON322806	2020/Oct	China	L	L	L	S	A	T	K	L	T	R
HN-064	CPV 2c	ON322807	2020/Oct	China	L	L	L	S	A	T	K	L	T	R
HN-065	CPV 2c	ON322808	2020/Oct	China	L	L	L	S	A	T	K	L	T	R
HN-066	CPV 2c	ON322809	2020/Oct	China	L	L	L	S	A	T	K	L	T	R
HN-068	CPV 2c	ON322810	2020/Aug	China	L	L	L	S	A	T	K	L	T	R
HN-069	CPV 2c	ON322811	2020/Sep	China	L	L	L	S	A	T	K	L	T	R
HN-070	CPV 2c	ON322812	2020/Aug	China	L	L	L	S	A	T	R	L	N	R
HN-071	CPV 2c	ON322813	2020/Sep	China	L	L	L	S	A	T	K	L	T	R
HN-072	CPV 2c	ON322814	2020/Sep	China	L	L	L	S	A	T	K	L	T	R
HN-073	CPV 2c	ON322815	2020/Sep	China	L	L	L	S	A	T	K	L	T	R
HN-074	CPV 2c	ON322816	2020/Sep	China	L	L	L	S	A	T	K	L	T	R
HN-075	CPV 2c	ON322817	2020/Sep	China	L	L	L	S	A	T	K	L	T	R
HN-076	CPV 2c	ON322818	2020/Sep	China	L	L	L	S	A	T	K	L	T	R
HN-077	CPV 2c	ON322819	2020/Oct	China	L	L	L	S	A	T	K	L	T	R
HN-078	CPV 2c	ON322820	2020/Aug	China	L	L	L	S	A	T	K	L	T	R
HN-079	CPV 2c	ON322821	2020/Nov	China	L	L	L	S	A	T	K	L	T	R
HN-080	CPV 2c	ON322822	2020/Nov	China	L	L	L	S	A	T	K	L	T	R
HN-081	CPV 2c	ON322823	2020/Nov	China	L	L	L	S	A	T	K	L	T	R
HN-082	CPV 2c	ON322824	2020/Nov	China	L	L	L	S	A	T	K	L	T	R
HN-084	CPV 2c	ON322825	2020/Nov	China	L	L	L	S	A	T	K	L	T	R
HN-085	CPV 2c	ON322826	2020/Nov	China	L	L	L	S	A	T	K	L	T	R
HN-086	CPV 2c	ON322827	2020/Nov	China	L	L	L	S	A	T	R	L	A	R
HN-087	CPV 2c	ON322828	2020/Nov	China	L	L	L	S	A	T	K	L	T	R
HN-088	CPV 2c	ON322829	2020/Nov	China	L	L	L	S	A	T	K	L	T	R
HN-089	New CPV 2a	ON322830	2020/Nov	China	L	L	L	S	T	T	R	L	A	R
HN-090	CPV 2c	ON322831	2020/Nov	China	L	L	L	S	A	T	K	L	T	R
HN-091	CPV 2c	ON322832	2020/Nov	China	L	L	L	S	A	T	K	L	T	R
HN-092	CPV 2c	ON322833	2020/Nov	China	L	L	L	S	A	T	K	L	T	R
HN-093	CPV 2c	ON322834	2020/Nov	China	L	L	L	S	A	T	K	L	A	R
HN-097	CPV 2c	ON322835	2020/Oct	China	L	L	L	S	A	T	K	L	T	R
HN-098	CPV 2c	ON322836	2020/Nov	China	L	L	L	S	A	T	K	L	T	R
HN-099	CPV 2c	ON322837	2021/Jan	China	L	L	L	S	A	T	K	L	T	R
HN-100	CPV 2c	ON322838	2021/Jan	China	L	L	L	S	A	T	K	L	T	R
HN-102	CPV 2c	ON322839	2021/Jan	China	L	L	L	S	A	T	K	L	T	R
HN-103	CPV 2c	ON322840	2021/Jan	China	L	L	L	S	A	T	K	L	T	R
HN-104	CPV 2c	ON322841	2021/Jan	China	L	L	L	S	A	T	K	L	T	R
HN-105	CPV 2c	ON322842	2021/Jan	China	L	L	L	S	A	T	K	L	T	R
HN-106	CPV 2c	ON322843	2021/Jan	China	L	L	L	S	A	T	K	L	T	R
HN-107	CPV 2c	ON322844	2021/Jan	China	L	L	L	S	A	T	K	L	T	R
HN-108	CPV 2c	ON322845	2021/Jan	China	L	L	L	S	A	T	K	L	T	R
HN-109	CPV 2c	ON322846	2021/Jan	China	L	L	L	S	A	T	K	L	T	R
HN-110	CPV 2c	ON322847	2020/Aug	China	L	L	L	S	A	T	K	L	T	R
HN-111	CPV 2c	ON322848	2020/Nov	China	L	L	L	S	A	T	K	L	T	R

a Shading indicates a mutation compared to the original CPV-2 (GenBank accession number M38245).

### Sequence comparison and amino acid sequence analysis of NS1.

Sequence comparisons revealed that the NS1 in this study exhibited a 98.8%–100% nucleotide identity and 99.0 to 100% amino acid identity. Compared with the NS1 of the traditional CPV-2 strain (M38245), most of the CPV-2c strains (*n* = 79) in our study presented with I60V, Y544F, E545V, and L630P mutations similar to the amino acid residues in the Chinese CPV-2c strain (MH476592) discovered in 2017 (Table 3). Both NS1 Y544F and E545V mutations occurred in most CPV-2c strains (*n* = 85) and a few new CPV-2a strains (*n* = 2). In contrast, the I60Vand L630P mutations were only identified in CPV-2c strains (*n* = 79). In addition, the E572K mutation was found in the new CPV-2a strains (HN-003, HN-013, HN-014, HN-015, HN-016, and HN-027) and CPV-2c strains (HN-019, HN-056, HN-057, HN-058, and HN-086). The NS1 amino acid sites (60I, 544Y, 545E, and 630L) of these strains were consistent with the original CPV-2 reference strain (M38245). Compared to other CPV-2 strains in Table 3, a unique amino acid mutation (E583K) was also observed in the new CPV-2a strains (HN-036 and HN-089) and CPV-2c strains (HN-038, HN-097, HN-104, HN-106, and HN-109). Notably, K19R and P599S in HN-036 and HN-041, respectively, were identified as two particular substitution positions in our study (Table 3). Other mutations (P590R, V596A, and N356S) appeared in some CPV 2c strains, and N624K existed in most new CPV 2a strains (*n* = 5).

**TABLE 3:** Amino acid mutations of the NS1 gene detected in this study^*a*^

Strain	Genetic type	GenBank accession no.	Yr	Origin	Amino acid position of NS1											
					19	60	356	544	545	572	583	590	596	599	624	630
Reference																
CPV-b	Original CPV 2	M38245	1978	USA	K	I	N	Y	E	E	E	P	V	P	N	L
CPV-15	CPV 2a	AY787926	1984	USA	K	I	N	Y	E	E	E	P	V	P	N	L
CPV-39	CPV 2b	AY787930	1984	USA	K	I	N	F	E	E	E	P	V	P	N	L
288-01	CPV 2c	MF177239	2001	Italy	K	I	N	Y	E	E	E	P	V	P	N	L
BJ03/17	New CPV 2a	MF134808	2017	China	K	I	N	Y	E	K	E	P	V	P	K	L
Canine/China/23	CPV 2c	MH476592	2017	China	K	V	N	F	V	E	E	P	V	P	N	P
HCM01	CPV 2c	MT106234	2017	Vietnam	K	V	N	F	V	E	E	P	V	P	N	P
This study																
HN-002	CPV 2c	ON322751	2020/June	China	K	V	N	F	V	E	E	P	V	P	N	P
HN-003	New CPV 2a	ON322752	2020/June	China	K	I	N	Y	E	K	E	P	V	P	N	L
HN-004	CPV 2c	ON322753	2020/June	China	K	V	N	F	V	E	E	P	V	P	N	P
HN-005	CPV 2c	ON322754	2020/June	China	K	V	N	F	V	E	E	P	V	P	N	P
HN-006	CPV 2c	ON322755	2020/June	China	K	V	N	F	V	E	E	P	V	P	N	P
HN-007	CPV 2c	ON322756	2020/June	China	K	V	N	F	V	E	E	P	V	P	N	P
HN-008	CPV 2c	ON322757	2020/June	China	K	V	N	F	V	E	E	P	V	P	N	P
HN-009	CPV 2c	ON322758	2020/July	China	K	V	N	F	V	E	E	P	V	P	N	P
HN-010	CPV 2c	ON322759	2020/July	China	K	V	N	F	V	E	E	P	V	P	N	P
HN-011	CPV 2c	ON322760	2020/July	China	K	V	N	F	V	E	E	P	V	P	N	P
HN-012	CPV 2c	ON322761	2020/Aug	China	K	V	N	F	V	E	E	P	V	P	N	P
HN-013	New CPV 2a	ON322762	2020/Aug	China	K	I	N	Y	E	K	E	P	V	P	K	L
HN-014	New CPV 2a	ON322763	2020/Aug	China	K	I	N	Y	E	K	E	P	V	P	K	L
HN-015	New CPV 2a	ON322764	2020/Aug	China	K	I	N	Y	E	K	E	P	V	P	K	L
HN-016	New CPV 2a	ON322765	2020/Aug	China	K	I	N	Y	E	K	E	P	V	P	K	L
HN-017	CPV 2c	ON322766	2020/July	China	K	V	N	F	V	E	E	P	V	P	N	P
HN-018	CPV 2c	ON322767	2020/Aug	China	K	V	N	F	V	E	E	P	V	P	N	P
HN-019	CPV 2c	ON322768	2020/Aug	China	K	I	N	Y	E	K	E	P	V	P	N	L
HN-021	CPV 2c	ON322769	2020/Aug	China	K	V	N	F	V	E	E	P	V	P	N	P
HN-022	CPV 2c	ON322770	2020/Aug	China	K	V	N	F	V	E	E	P	V	P	N	P
HN-023	CPV 2c	ON322771	2020/Aug	China	K	V	N	F	V	E	E	P	V	P	N	P
HN-024	CPV 2c	ON322772	2020/Aug	China	K	V	N	F	V	E	E	P	V	P	N	P
HN-025	CPV 2c	ON322773	2020/Aug	China	K	V	N	F	V	E	E	P	V	P	N	P
HN-026	CPV 2c	ON322774	2020/July	China	K	V	N	F	V	E	E	P	V	P	N	P
HN-027	New CPV 2a	ON322775	2020/July	China	K	I	N	Y	E	K	E	P	V	P	K	L
HN-028	CPV 2c	ON322776	2020/Aug	China	K	V	N	F	V	E	E	P	V	P	N	P
HN-029	CPV 2c	ON322777	2020/Aug	China	K	V	N	F	V	E	E	P	V	P	N	P
HN-030	CPV 2c	ON322778	2020/Aug	China	K	V	N	F	V	E	E	P	V	P	N	P
HN-031	CPV 2c	ON322779	2020/Aug	China	K	V	N	F	V	E	E	P	V	P	N	P
HN-033	CPV 2c	ON322780	2020/Aug	China	K	V	N	F	V	E	E	P	V	P	N	P
HN-034	CPV 2c	ON322781	2020/Aug	China	K	V	N	F	V	E	E	P	V	P	N	P
HN-035	CPV 2c	ON322782	2020/July	China	K	V	N	F	V	E	E	P	V	P	N	P
HN-036	New CPV 2a	ON322783	2020/Aug	China	R	I	N	F	V	E	K	P	V	P	N	L
HN-038	CPV 2c	ON322784	2020/Aug	China	K	I	N	F	V	E	K	P	V	P	N	L
HN-039	CPV 2c	ON322785	2020/Aug	China	K	V	N	F	V	E	E	P	V	P	N	P
HN-040	CPV 2c	ON322786	2020/Aug	China	K	V	N	F	V	E	E	P	V	P	N	P
HN-041	CPV 2c	ON322787	2020/Aug	China	K	V	N	F	V	E	E	P	V	S	N	P
HN-042	CPV 2c	ON322788	2020/Aug	China	K	V	N	F	V	E	E	P	V	P	N	P
HN-043	CPV 2c	ON322789	2020/Aug	China	K	V	N	F	V	E	E	P	V	P	N	P
HN-044	CPV 2c	ON322790	2020/Aug	China	K	V	N	F	V	E	E	P	V	P	N	P
HN-045	CPV 2c	ON322791	2020/Aug	China	K	V	N	F	V	E	E	P	V	P	N	P
HN-046	CPV 2c	ON322792	2020/Aug	China	K	V	S	F	V	E	E	P	V	P	N	P
HN-047	CPV 2c	ON322793	2020/Aug	China	K	V	N	F	V	E	E	P	V	P	N	P
HN-048	CPV 2c	ON322794	2020/Aug	China	K	V	N	F	V	E	E	P	V	P	N	P
HN-049	CPV 2c	ON322795	2020/Sep	China	K	V	N	F	V	E	E	P	V	P	N	P
HN-050	CPV 2c	ON322796	2020/Sep	China	K	I	N	F	V	E	E	P	V	P	N	P
HN-051	CPV 2c	ON322797	2020/Sep	China	K	V	N	F	V	E	E	R	V	P	N	P
HN-055	CPV 2c	ON322798	2020/Sep	China	K	V	N	F	V	E	E	P	V	P	N	P
HN-056	CPV 2c	ON322799	2020/Oct	China	K	I	N	Y	E	K	E	P	V	P	N	L
HN-057	CPV 2c	ON322800	2020/Oct	China	K	I	N	Y	E	K	E	P	V	P	N	L
HN-058	CPV 2c	ON322801	2020/Oct	China	K	I	N	Y	E	K	E	P	V	P	N	L
HN-059	CPV 2c	ON322802	2020/Oct	China	K	V	N	F	V	E	E	P	V	P	N	P
HN-060	CPV 2c	ON322803	2020/Oct	China	K	V	N	F	V	E	E	P	V	P	N	P
HN-061	CPV 2c	ON322804	2020/Oct	China	K	V	N	F	V	E	E	P	V	P	N	P
HN-062	CPV 2c	ON322805	2020/Oct	China	K	V	N	F	V	E	E	P	V	P	N	P
HN-063	CPV 2c	ON322806	2020/Oct	China	K	V	N	F	V	E	E	P	V	P	N	P
HN-064	CPV 2c	ON322807	2020/Oct	China	K	V	N	F	V	E	E	R	V	P	N	P
HN-065	CPV 2c	ON322808	2020/Oct	China	K	V	N	F	V	E	E	P	V	P	N	P
HN-066	CPV 2c	ON322809	2020/Oct	China	K	V	N	F	V	E	E	P	V	P	N	P
HN-068	CPV 2c	ON322810	2020/Aug	China	K	V	N	F	V	E	E	P	V	P	N	P
HN-069	CPV 2c	ON322811	2020/Sep	China	K	V	N	F	V	E	E	P	V	P	N	P
HN-070	CPV 2c	ON322812	2020/Aug	China	K	V	N	F	V	E	E	P	V	P	N	P
HN-071	CPV 2c	ON322813	2020/Sep	China	K	V	N	F	V	E	E	P	V	P	N	P
HN-072	CPV 2c	ON322814	2020/Sep	China	K	V	N	F	V	E	E	P	A	P	N	P
HN-073	CPV 2c	ON322815	2020/Sep	China	K	V	N	F	V	E	E	P	A	P	N	P
HN-074	CPV 2c	ON322816	2020/Sep	China	K	V	N	F	V	E	E	P	V	P	N	P
HN-075	CPV 2c	ON322817	2020/Sep	China	K	V	N	F	V	E	E	P	A	P	N	P
HN-076	CPV 2c	ON322818	2020/Sep	China	K	V	N	F	V	E	E	P	A	P	N	P
HN-077	CPV 2c	ON322819	2020/Oct	China	K	V	N	F	V	E	E	P	V	P	N	P
HN-078	CPV 2c	ON322820	2020/Aug	China	K	V	N	F	V	E	E	P	V	P	N	P
HN-079	CPV 2c	ON322821	2020/Nov	China	K	V	N	F	V	E	E	P	V	P	N	P
HN-080	CPV 2c	ON322822	2020/Nov	China	K	V	N	F	V	E	E	P	V	P	N	P
HN-081	CPV 2c	ON322823	2020/Nov	China	K	V	N	F	V	E	E	P	V	P	N	P
HN-082	CPV 2c	ON322824	2020/Nov	China	K	V	N	F	V	E	E	P	V	P	N	P
HN-084	CPV 2c	ON322825	2020/Nov	China	K	V	N	F	V	E	E	P	V	P	N	P
HN-085	CPV 2c	ON322826	2020/Nov	China	K	V	N	F	V	E	E	P	V	P	N	P
HN-086	CPV 2c	ON322827	2020/Nov	China	K	I	N	Y	E	K	E	P	V	P	N	L
HN-087	CPV 2c	ON322828	2020/Nov	China	K	V	N	F	V	E	E	P	V	P	N	P
HN-088	CPV 2c	ON322829	2020/Nov	China	K	V	N	F	V	E	E	P	V	P	N	P
HN-089	New CPV 2a	ON322830	2020/Nov	China	K	I	N	F	V	E	K	P	V	P	N	L
HN-090	CPV 2c	ON322831	2020/Nov	China	K	V	N	F	V	E	E	P	V	P	N	P
HN-091	CPV 2c	ON322832	2020/Nov	China	K	V	N	F	V	E	E	P	V	P	N	P
HN-092	CPV 2c	ON322833	2020/Nov	China	K	V	N	F	V	E	E	P	V	P	N	P
HN-093	CPV 2c	ON322834	2020/Nov	China	K	V	N	F	V	E	E	P	V	P	N	P
HN-097	CPV 2c	ON322835	2020/Oct	China	K	I	N	F	V	E	K	P	V	P	N	L
HN-098	CPV 2c	ON322836	2020/Nov	China	K	V	N	F	V	E	E	P	V	P	N	P
HN-099	CPV 2c	ON322837	2021/Jan	China	K	V	N	F	V	E	E	P	V	P	N	P
HN-100	CPV 2c	ON322838	2021/Jan	China	K	V	N	F	V	E	E	P	V	P	N	P
HN-102	CPV 2c	ON322839	2021/Jan	China	K	V	N	F	V	E	E	P	V	P	N	P
HN-103	CPV 2c	ON322840	2021/Jan	China	K	V	N	F	V	E	E	P	V	P	N	P
HN-104	CPV 2c	ON322841	2021/Jan	China	K	I	N	F	V	E	K	P	V	P	N	L
HN-105	CPV 2c	ON322842	2021/Jan	China	K	V	N	F	V	E	E	P	V	P	N	P
HN-106	CPV 2c	ON322843	2021/Jan	China	K	I	N	F	V	E	K	P	V	P	N	L
HN-107	CPV 2c	ON322844	2021/Jan	China	K	V	N	F	V	E	E	P	V	P	N	P
HN-108	CPV 2c	ON322845	2021/Jan	China	K	V	N	F	V	E	E	P	V	P	N	P
HN-109	CPV 2c	ON322846	2021/Jan	China	K	I	N	F	V	E	K	P	V	P	N	L
HN-110	CPV 2c	ON322847	2020/Aug	China	K	V	N	F	V	E	E	R	V	P	N	P
HN-111	CPV 2c	ON322848	2020/Nov	China	K	V	N	F	V	E	E	P	V	P	N	P

a Shading indicates a mutation compared to the original CPV-2 (GenBank accession number M38245).

### Specific amino acid mutations in the NS2 proteins.

The amino acid variants K19R and I60V of the NS2 protein were also located at the same site in the NS1-encoding sequence. R92K mutation in the NS2 protein was observed in five CPV-2c strains and two new CPV 2a strains (Table 4). Compared to the original CPV-2 (M38245), the NS2 S134A mutation was observed in five new CPV-2a (HN-013, HN-014, HN-015, HN-016, and HN- 027) strains, but was not observed in the CPV-2c strains (Table 4). All the new CPV 2a and CPV-2c strains presented the NS2 D151N and M152V mutations which were similar to the residues in the Chinese CPV-2c (MH476592) and new CPV-2a (MF134808) strains (Table 4). Compared to all the reference strains, a unique N155D mutation was discovered in the NS2 protein of four CPV-2c strains (HN-019, HN-056, HN-057, and HN-058). Four additional amino acid changes in the NS2-encoding sequences were exhibited: T94A, E101K, N107H, and S108F (Table 4).

**TABLE 4:** Amino acid mutations of the NS2 gene detected in this study^*a*^

Strain	Genetic type	GenBank accession no.	Yr	Origin	Amino acid position of NS2										
					19	60	92	94	101	107	108	134	151	152	155
Reference															
CPV-b	Original CPV2	M38245	1978	USA	K	I	R	T	E	N	S	S	D	M	N
CPV-15	CPV 2a	M24003	1984	USA	K	I	R	T	E	N	S	S	D	M	N
CPV-39	CPV 2b	M74849	1984	USA	K	I	R	T	E	N	S	S	D	V	N
288-01	CPV 2c	MF177239	2001	Italy	K	I	R	A	E	N	S	S	N	V	N
BJ03/17	New CPV 2a	MF134808	2017	China	K	I	R	A	E	N	S	A	N	V	N
Canine/China/23	CPV 2c	MH476592	2017	China	K	V	R	T	E	N	S	S	N	V	N
HCM01	CPV 2c	MT106234	2017	Vietnam	K	V	R	T	E	N	S	S	N	V	N
This study															
HN-002	CPV 2c	ON322751	2020/June	China	K	V	R	T	E	N	S	S	N	V	N
HN-003	New CPV 2a	ON322752	2020/June	China	K	I	R	A	E	N	S	S	N	V	N
HN-004	CPV 2c	ON322753	2020/June	China	K	V	R	T	E	N	S	S	N	V	N
HN-005	CPV 2c	ON322754	2020/June	China	K	V	R	T	E	N	S	S	N	V	N
HN-006	CPV 2c	ON322755	2020/June	China	K	V	R	T	E	N	S	S	N	V	N
HN-007	CPV 2c	ON322756	2020/June	China	K	V	R	T	E	N	S	S	N	V	N
HN-008	CPV 2c	ON322757	2020/June	China	K	V	R	T	E	N	S	S	N	V	N
HN-009	CPV 2c	ON322758	2020/July	China	K	V	R	T	E	N	S	S	N	V	N
HN-010	CPV 2c	ON322759	2020/July	China	K	V	R	T	E	N	S	S	N	V	N
HN-011	CPV 2c	ON322760	2020/July	China	K	V	R	T	E	N	S	S	N	V	N
HN-012	CPV 2c	ON322761	2020/Aug	China	K	V	R	T	E	N	S	S	N	V	N
HN-013	New CPV 2a	ON322762	2020/Aug	China	K	I	R	A	E	N	S	A	N	V	N
HN-014	New CPV 2a	ON322763	2020/Aug	China	K	I	R	A	E	N	S	A	N	V	N
HN-015	New CPV 2a	ON322764	2020/Aug	China	K	I	R	A	E	N	S	A	N	V	N
HN-016	New CPV 2a	ON322765	2020/Aug	China	K	I	R	A	E	N	S	A	N	V	N
HN-017	CPV 2c	ON322766	2020/July	China	K	V	R	T	E	H	S	S	N	V	N
HN-018	CPV 2c	ON322767	2020/Aug	China	K	V	R	T	E	N	S	S	N	V	N
HN-019	CPV 2c	ON322768	2020/Aug	China	K	I	R	A	E	N	S	S	N	V	D
HN-021	CPV 2c	ON322769	2020/Aug	China	K	V	R	T	E	N	S	S	N	V	N
HN-022	CPV 2c	ON322770	2020/Aug	China	K	V	R	T	E	N	S	S	N	V	N
HN-023	CPV 2c	ON322771	2020/Aug	China	K	V	R	T	E	N	S	S	N	V	N
HN-024	CPV 2c	ON322772	2020/Aug	China	K	V	R	T	E	N	S	S	N	V	N
HN-025	CPV 2c	ON322773	2020/Aug	China	K	V	R	T	E	N	S	S	N	V	N
HN-026	CPV 2c	ON322774	2020/July	China	K	V	R	T	E	N	S	S	N	V	N
HN-027	New CPV 2a	ON322775	2020/July	China	K	I	R	A	E	N	S	A	N	V	N
HN-028	CPV 2c	ON322776	2020/Aug	China	K	V	R	T	E	N	S	S	N	V	N
HN-029	CPV 2c	ON322777	2020/Aug	China	K	V	R	T	E	N	S	S	N	V	N
HN-030	CPV 2c	ON322778	2020/Aug	China	K	V	R	T	E	N	S	S	N	V	N
HN-031	CPV 2c	ON322779	2020/Aug	China	K	V	R	T	E	N	S	S	N	V	N
HN-033	CPV 2c	ON322780	2020/Aug	China	K	V	R	T	E	N	S	S	N	V	N
HN-034	CPV 2c	ON322781	2020/Aug	China	K	V	R	T	E	N	S	S	N	V	N
HN-035	CPV 2c	ON322782	2020/July	China	K	V	R	T	E	N	S	S	N	V	N
HN-036	New CPV 2a	ON322783	2020/Aug	China	R	I	K	T	E	N	S	S	N	V	N
HN-038	CPV 2c	ON322784	2020/Aug	China	K	I	K	T	E	N	S	S	N	V	N
HN-039	CPV 2c	ON322785	2020/Aug	China	K	V	R	T	E	N	S	S	N	V	N
HN-040	CPV 2c	ON322786	2020/Aug	China	K	V	R	T	E	N	S	S	N	V	N
HN-041	CPV 2c	ON322787	2020/Aug	China	K	V	R	T	E	N	F	S	N	V	N
HN-042	CPV 2c	ON322788	2020/Aug	China	K	V	R	T	E	N	S	S	N	V	N
HN-043	CPV 2c	ON322789	2020/Aug	China	K	V	R	T	E	N	S	S	N	V	N
HN-044	CPV 2c	ON322790	2020/Aug	China	K	V	R	T	E	N	S	S	N	V	N
HN-045	CPV 2c	ON322791	2020/Aug	China	K	V	R	T	E	N	S	S	N	V	N
HN-046	CPV 2c	ON322792	2020/Aug	China	K	V	R	T	K	N	S	S	N	V	N
HN-047	CPV 2c	ON322793	2020/Aug	China	K	V	R	T	E	N	S	S	N	V	N
HN-048	CPV 2c	ON322794	2020/Aug	China	K	V	R	T	E	N	S	S	N	V	N
HN-049	CPV 2c	ON322795	2020/Sep	China	K	V	R	T	E	N	S	S	N	V	N
HN-050	CPV 2c	ON322796	2020/Sep	China	K	I	R	T	E	N	S	S	N	V	N
HN-051	CPV 2c	ON322797	2020/Sep	China	K	V	R	T	E	N	S	S	N	V	N
HN-055	CPV 2c	ON322798	2020/Sep	China	K	V	R	T	E	N	S	S	N	V	N
HN-056	CPV 2c	ON322799	2020/Oct	China	K	I	R	A	E	N	S	S	N	V	D
HN-057	CPV 2c	ON322800	2020/Oct	China	K	I	R	A	E	N	S	S	N	V	D
HN-058	CPV 2c	ON322801	2020/Oct	China	K	I	R	A	E	N	S	S	N	V	D
HN-059	CPV 2c	ON322802	2020/Oct	China	K	V	R	T	E	H	S	S	N	V	N
HN-060	CPV 2c	ON322803	2020/Oct	China	K	V	R	T	E	N	S	S	N	V	N
HN-061	CPV 2c	ON322804	2020/Oct	China	K	V	R	T	E	N	S	S	N	V	N
HN-062	CPV 2c	ON322805	2020/Oct	China	K	V	R	T	E	N	S	S	N	V	N
HN-063	CPV 2c	ON322806	2020/Oct	China	K	V	R	T	E	N	S	S	N	V	N
HN-064	CPV 2c	ON322807	2020/Oct	China	K	V	R	T	E	N	S	S	N	V	N
HN-065	CPV 2c	ON322808	2020/Oct	China	K	V	R	T	E	N	S	S	N	V	N
HN-066	CPV 2c	ON322809	2020/Oct	China	K	V	R	T	E	N	S	S	N	V	N
HN-068	CPV 2c	ON322810	2020/Aug	China	K	V	R	T	E	N	S	S	N	V	N
HN-069	CPV 2c	ON322811	2020/Sep	China	K	V	R	T	E	N	S	S	N	V	N
HN-070	CPV 2c	ON322812	2020/Aug	China	K	V	R	T	E	N	S	S	N	V	N
HN-071	CPV 2c	ON322813	2020/Sep	China	K	V	R	T	E	N	S	S	N	V	N
HN-072	CPV 2c	ON322814	2020/Sep	China	K	V	R	T	E	N	S	S	N	V	N
HN-073	CPV 2c	ON322815	2020/Sep	China	K	V	R	T	E	N	S	S	N	V	N
HN-074	CPV 2c	ON322816	2020/Sep	China	K	V	R	T	E	N	S	S	N	V	N
HN-075	CPV 2c	ON322817	2020/Sep	China	K	V	R	T	E	N	S	S	N	V	N
HN-076	CPV 2c	ON322818	2020/Sep	China	K	V	R	T	E	N	S	S	N	V	N
HN-077	CPV 2c	ON322819	2020/Oct	China	K	V	R	T	E	N	S	S	N	V	N
HN-078	CPV 2c	ON322820	2020/Aug	China	K	V	R	T	E	N	S	S	N	V	N
HN-079	CPV 2c	ON322821	2020/Nov	China	K	V	R	T	E	N	S	S	N	V	N
HN-080	CPV 2c	ON322822	2020/Nov	China	K	V	R	T	E	N	S	S	N	V	N
HN-081	CPV 2c	ON322823	2020/Nov	China	K	V	R	T	E	N	S	S	N	V	N
HN-082	CPV 2c	ON322824	2020/Nov	China	K	V	R	T	E	N	S	S	N	V	N
HN-084	CPV 2c	ON322825	2020/Nov	China	K	V	R	T	E	N	S	S	N	V	N
HN-085	CPV 2c	ON322826	2020/Nov	China	K	V	R	T	K	N	S	S	N	V	N
HN-086	CPV 2c	ON322827	2020/Nov	China	K	I	R	A	E	N	S	S	N	V	N
HN-087	CPV 2c	ON322828	2020/Nov	China	K	V	R	T	E	N	S	S	N	V	N
HN-088	CPV 2c	ON322829	2020/Nov	China	K	V	R	T	E	N	S	S	N	V	N
HN-089	New CPV 2a	ON322830	2020/Nov	China	K	I	K	T	E	N	S	S	N	V	N
HN-090	CPV 2c	ON322831	2020/Nov	China	K	V	R	T	E	N	S	S	N	V	N
HN-091	CPV 2c	ON322832	2020/Nov	China	K	V	R	T	E	N	S	S	N	V	N
HN-092	CPV 2c	ON322833	2020/Nov	China	K	V	R	T	E	N	S	S	N	V	N
HN-093	CPV 2c	ON322834	2020/Nov	China	K	V	R	T	E	N	S	S	N	V	N
HN-097	CPV 2c	ON322835	2020/Oct	China	K	I	K	T	E	N	S	S	N	V	N
HN-098	CPV 2c	ON322836	2020/Nov	China	K	V	R	T	E	N	S	S	N	V	N
HN-099	CPV 2c	ON322837	2021/Jan	China	K	V	R	T	E	N	S	S	N	V	N
HN-100	CPV 2c	ON322838	2021/Jan	China	K	V	R	T	E	N	S	S	N	V	N
HN-102	CPV 2c	ON322839	2021/Jan	China	K	V	R	T	E	N	S	S	N	V	N
HN-103	CPV 2c	ON322840	2021/Jan	China	K	V	R	T	E	N	S	S	N	V	N
HN-104	CPV 2c	ON322841	2021/Jan	China	K	I	K	T	E	N	S	S	N	V	N
HN-105	CPV 2c	ON322842	2021/Jan	China	K	V	R	T	E	N	S	S	N	V	N
HN-106	CPV 2c	ON322843	2021/Jan	China	K	I	K	T	E	N	S	S	N	V	N
HN-107	CPV 2c	ON322844	2021/Jan	China	K	V	R	T	E	N	S	S	N	V	N
HN-108	CPV 2c	ON322845	2021/Jan	China	K	V	R	T	E	N	S	S	N	V	N
HN-109	CPV 2c	ON322846	2021/Jan	China	K	I	K	T	E	N	S	S	N	V	N
HN-110	CPV 2c	ON322847	2020/Aug	China	K	V	R	T	E	N	S	S	N	V	N
HN-111	CPV 2c	ON322848	2020/Nov	China	K	V	R	T	E	N	S	S	N	V	N

a Shading indicates a mutation compared to the original CPV-2 (GenBank accession number M38245).

### Phylogenetic analysis of the VP2 and NS1 amino acid sequences.

**FIG 1:**
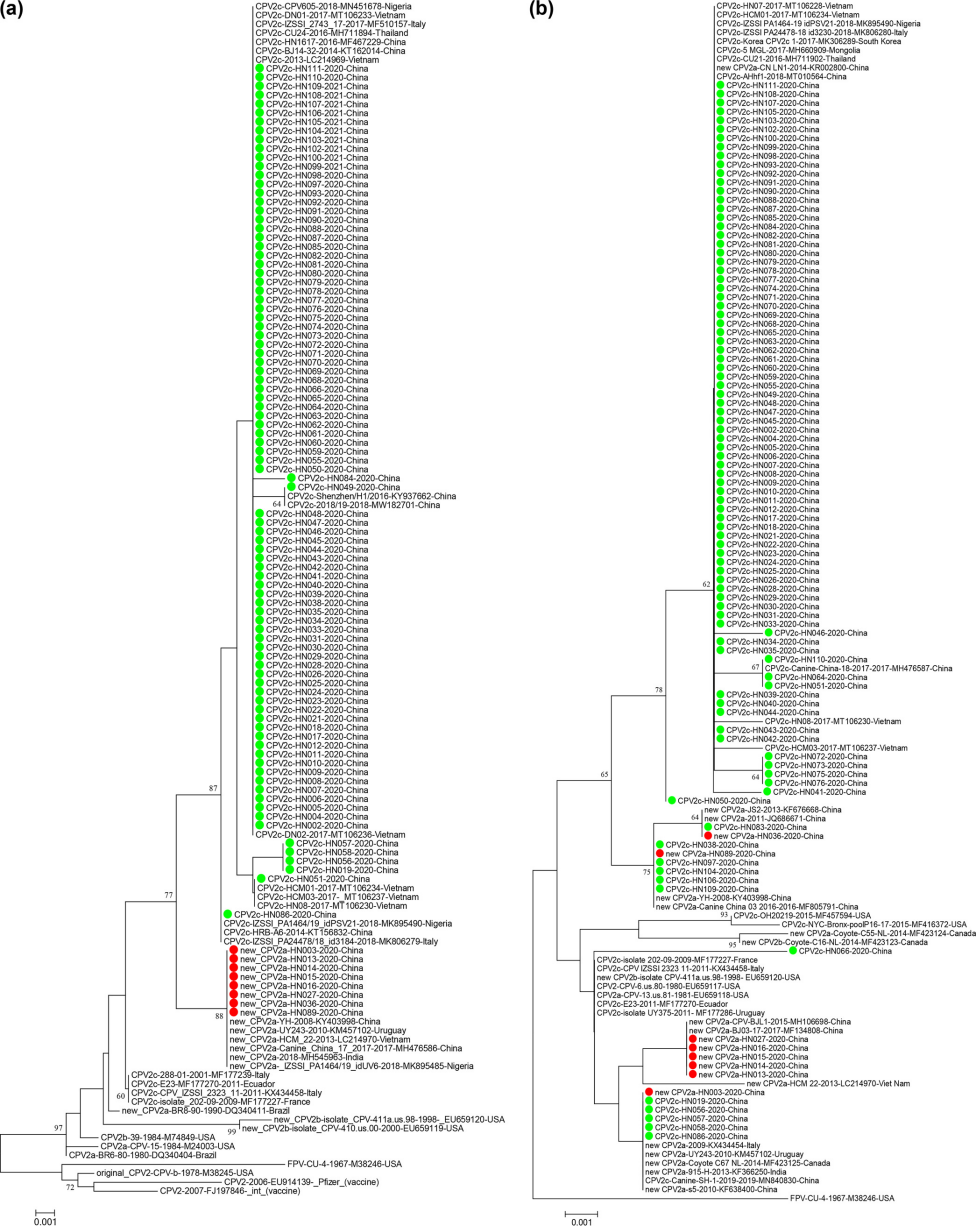
Neighbor-joining trees based on the deduced complete VP2 (a) and NS1 (b) amino acid sequences of canine parvovirus type 2 (CPV-2). Bootstrap values at the nodes are based on 1,000 replicates and bootstrap values greater than 60 are shown. Each sequence is indicated with the virus type (FPV, feline panleukopenia virus; CPV, canine parvovirus) or variant (CPV-2, CPV-2a, CPV-2b, new CPV-2a, new CPV-2b, and CPV-2c), strain/isolate name, year of collection, accession number, and country of collection. The Chinese CPV-2c and new CPV-2a strains in this study are marked with green dots and red dots, respectively.

Neighbor-joining trees based on the VP2 amino acid sequences of CPV-2 strains (98 strains in our study and a data set of 36 CPV-2 VP2 sequences available in GenBank) indicated that both the CPV-2c and new CPV-2a strains in this study were closely clustered with the Asian CPV-2 strains (Fig. 1a). The CPV-2c and new CPV-2a strains sequenced in this study were located in two large different branches. The new CPV-2a strains (HN-003, HN-013, HN-014, HN-015, HN-016, HN-027, HN-036, and HN-089) were clustered together in the same branch with the new CPV-2a reference strains (KY403998, LC214970, MH476586, and MH545963). However, the CPV-2c strains were clustered in several different branches. Many CPV-2c strains (*n* = 83) with 5G and 447I in VP2 proteins were clustered in a large branch with the reference CPV-2c mutants DN01-2017-MT106233-Vietnam, HN1617-2016-MF467229-China, and CU24-2016-MH711894-Thailand. The CPV-2c HN-051 strain with A5G and I447M mutations in the VP2 protein was clustered in an identical small branch with the latest Vietnam CPV-2c mutant (MK357726, MT106234, and MT106230). However, the CPV-2c variants, HN019, HN056, HN057, and HN058 with 5A and 447M in the VP2 proteins were located in a large cluster but on separate secondary branches from the latest Vietnamese CPV-2c mutants (MK357726, MT106234, and MT106230). Among the CPV-2c strains in this study, only one strain (HN086) with VP2 5A was found to be evolutionarily close to the traditional genotype CPV-2c reference strains, i.e., KT156832, MK895490, and MK806279.

A phylogenetic analysis inferred from the NS1 sequences also showed that the CPV-2 strains in our study were more closely related to the CPV-2 strains of Asian origin (Fig. 1b). CPV-2 variant with the same genotype appeared to have a closer NS1 evolutionary relationship. As shown in Fig. 1b, most NS1 sequences of the CPV-2c mutants in this study were clustered in a large branch with the Asian CPV-2c reference strains (MT106228, MK306289, MH660909, and MT010564). The new CPV-2a strains HN013, HN014, HN015, HN016, and HN027 were clustered together with the new CPV-2a Chinese reference strains MH106698 and MF134808. Nevertheless, the CPV-2c mutant strains HN019, HN056, HN057, HN058, and HN086 were clustered in the same branch with the new CPV-2a reference strains (KX434454, KM457102, MF423125, KF366250, and KF638400), which was inconsistent with the VP2 phylogenetic tree. This finding suggests that recombination may have occurred among the CPV-2 strains.

### Epidemiological changes of the different CPV-2 variants in China.

**FIG 2:**
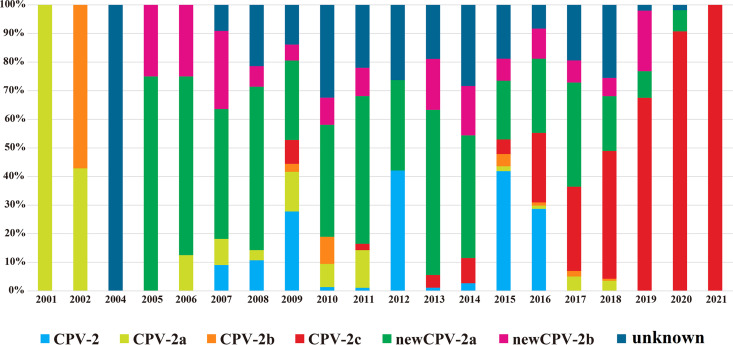
Prevalence of CPV-2 genotypes circulating in China from 2001 to 2021. The genotypes were confirmed by analyzing VP2 amino acid sequences from this study and the Chinese strains available in the GenBank database (accessed June 2021).

To clarify the evolution of CPV-2 strains in China, the VP2 sequences of 724 CPV-2 strains in China (including the sequences obtained in this study) were classified and analyzed according to the collection date and variant type (Fig. 2). The results showed that CPV-2a and CPV-2b were the main circulating CPV-2 strains in China from 2001 to 2002. From 2005 to 2014, new CPV-2a was the dominant genotype and the prevalence of new CPV-2a showed a downward trend after 2017. The prevalence of new CPV-2b virus strain remained low and relatively stable from 2005 to 2019, which is similar to the results of a previous study (41). CPV-2c was first reported in China in 2009 (33) but subsequently exhibited a low prevalence in China from 2009 to 2015. However, since 2017, the prevalence rate of CPV-2c in China increased annually and exceeded the prevalence of the new CPV-2a in 2018, and gradually became the primary CPV-2 variant in China. These results are based on incomplete GenBank submissions and related publications, which may have an impact on an analysis of the results.

### Population dynamic analyses of CPV-2 in China.

**FIG 3:**
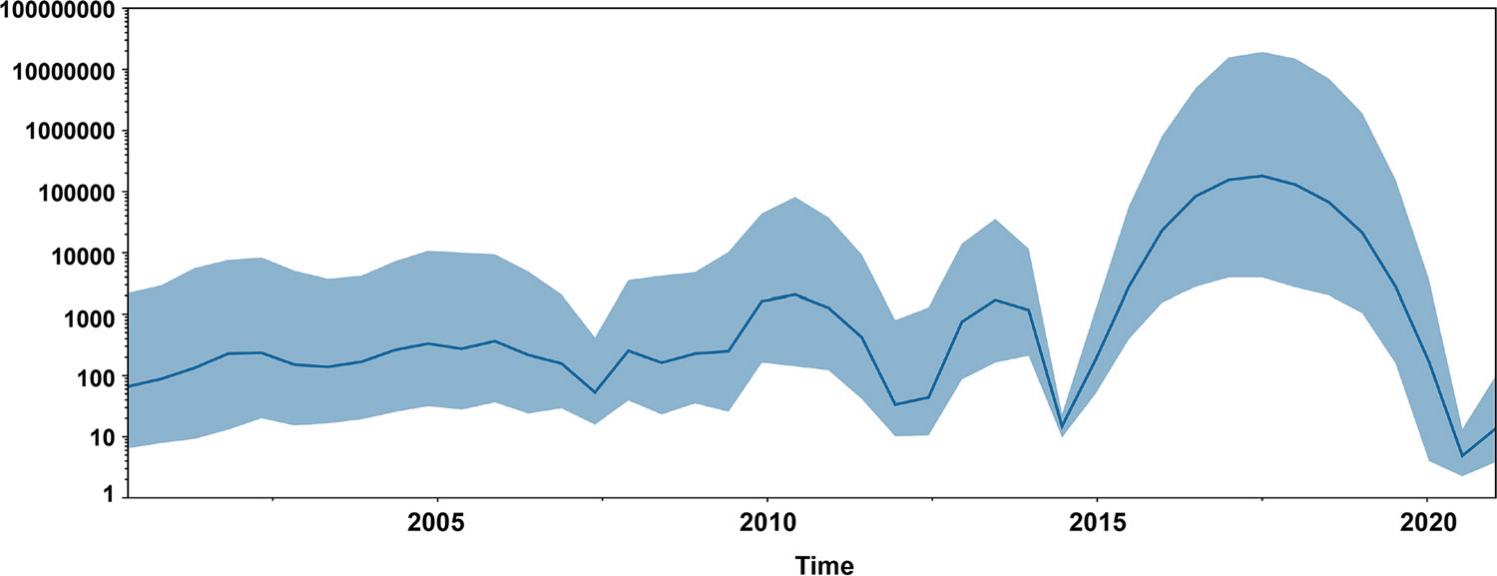
Bayesian Skygrid coalescent (BSG) of the complete VP2 gene of CPV-2 in China. The dark blue line indicates the mean value of genetic diversity, and the light blue shading shows the 95% confidence interval.

Our temporal signal evaluation indicated that the heterochronic sampling dates could be adapted for molecular clock calibration for BEAST analysis (*r*^2^ = 0.34) (see Fig. S1). The population size of CPV-2 was estimated by the BSG based on the CVP-2 VP2 gene. The Bayesian skyline plot revealed a low and stable growth trend in population size of CPV-2 until about 2006. Moreover, there were three obvious waves in the population size of CPV-2 between 2007 and 2020. The first wave commenced around 2009, and the CPV-2c strain was first detected in China (33). When the second significant wave appeared around 2013, new CPV-2a (Ser297Ala) and new CPV-2b (Ser297Ala) were the dominant strains, followed by CPV-2c (Fig. 3) (20, 28, 38). The population increased sharply from 2014, peaked in 2017, and then declined until a new inflection point of growth occurred after 2020.

### MCC time-resolved tree analyses of CPV-2 in China.

**FIG 4:**
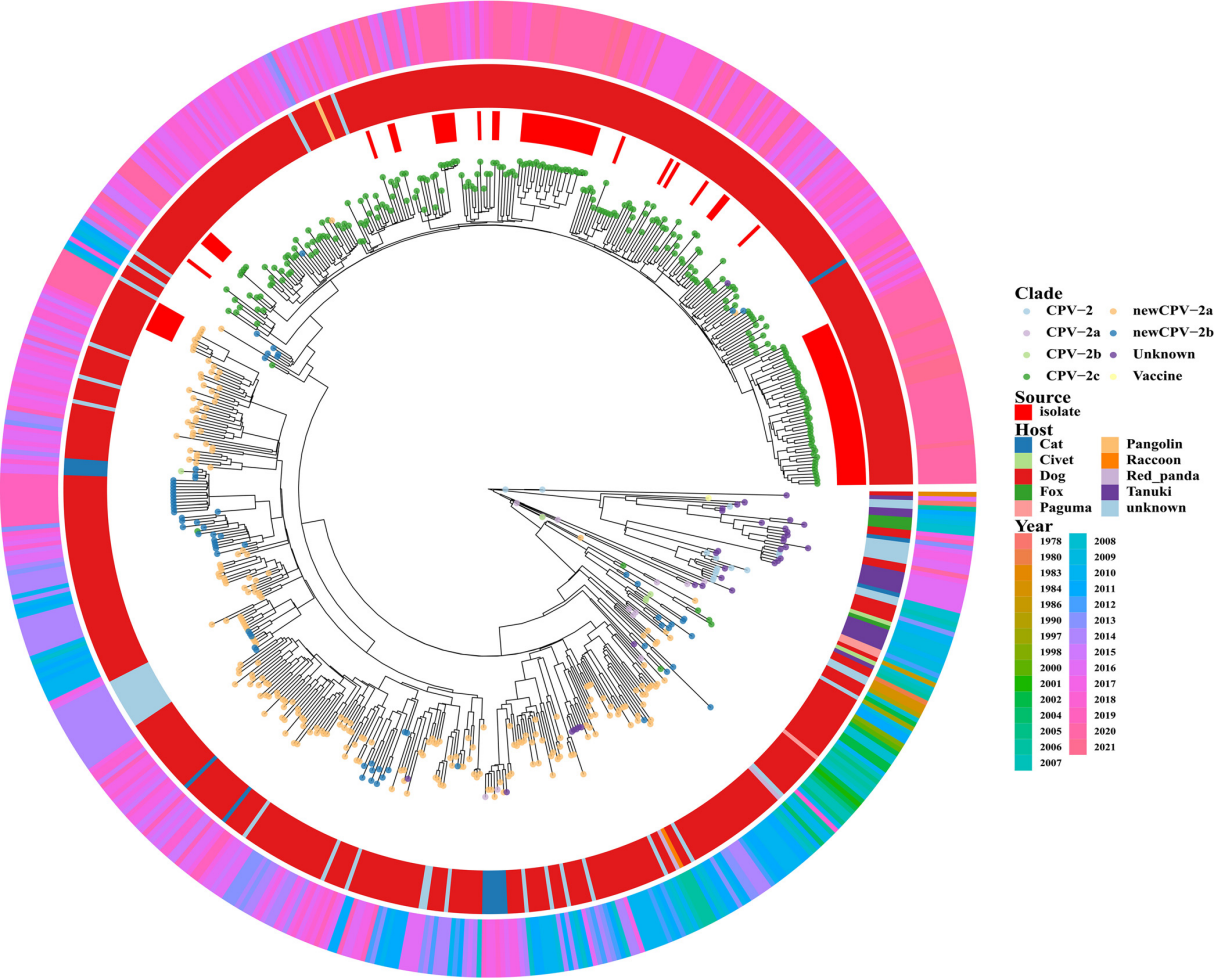
Maximum clade credibility (MCC) tree of Chinese CPV-2 inferred from VP2 sequences collected from GenBank and in this study. The MCC tree was obtained using TreeAnnotator (part of BEAST v1.10.4). The ggtree and ggplot2 packages in R (v4.0.5) were used to visualize the tree. The outside circle (gradient color) represents the year in which the strain sequence was detected. The middle circle (different colors) represents the hosts of the strains. The incomplete inside circle (red) indicates the sequences of this study. Different colored solid dots at the ends of the clade represent different CPV-2 genotypes.

The MCC tree was generated based on the VP2 gene of all Chinese strains available in the GenBank database (accessed June 2021) and the strains sequenced in this study (Fig. 4). Recently, there has been a shift in the predominant CPV-2 genotype from new CPV-2a and new CPV-2b to CPV-2c. Moreover, as depicted in Fig. 4, the initial CPV-2 genotype has a wider host range, followed by new CPV-2a and new CPV-2b, and eventually to CPV-2c. The majority of CPV-2c strains were derived from dogs. Therefore, CPV-2 may be gradually evolving toward becoming more adapted to infect dogs.

### Adaptation analyses.

Positive selection sites were evaluated using the MEME methods. MEME analysis showed that 17 positive selection sites (sites 5, 63, 91, 92, 119, 173, 174, 177, 188, 195, 270, 324, 370, 419, 426, 457, and 571) were identified in the VP2 protein of CPV-2 (see Table S3). We evaluated parallel evolution by examining amino acid substitutions along the VP2 phylogenetic tree of all CPV-2 strains from China in GenBank and our study. The CPV-2 VP2 protein had 10 amino acids that evolved in parallel (A5G, P13S, L87M, I219V, F267Y, S297A, Q370R, D375N, N426D/D426E, and I447M). In addition, E/N426D was detected as part of convergent evolution. Notably, three sites—5, 370, and 426—were both under positive selection pressure and parallel evolution.

## DISCUSSION

In China, CPV-2 infection was first described in 1982, after which various mutants of CPV-2 were subsequently detected in the canine population. Previous studies have demonstrated that the new CPV-2a was considered to be the predominant genotype in China for a long duration, cocirculating with the other two mutant genotypes (new CPV-2b and CPV-2c) (45–47). In our study, 88.29% (98/111) of clinical samples were CPV-2 positive, indicating that CPV-2 was widespread among the dog population in Henan province from 2022 to 2021. The detection of clinical samples observed that a certain proportion of CoV (15.3%, 15/98) or CAV-II (9.2%, 9/98) coinfections occurred in CPV-2 infection cases in Henan province, China. Therefore, CoV or CAV-II mixed infection should also be fully considered in the clinical diagnosis and treatment of CPV-2 cases.

Most of the CPV-2 strains obtained in this study were CPV-2c (*n* = 90), a few samples were new CPV-2a (*n* = 8), and no other CPV-2 genotypes were detected, indicating that CPV-2c had become the main epidemic genotype in Henan province, China. Combined with the VP2 sequences available in the NCBI database and VP2 gene sequences obtained in our study, the evolution of CPV-2 strains in China was further analyzed in this study. Our results showed that the prevalence of CPV-2c in China increased annually from about 2017 and exceeded the new CPV-2a strain in 2018, suggesting that CPV-2c may become the primary CPV-2 variant in China (Fig. 2).

VP2 is the major structural protein of CPV-2, which induces the production of protective antibodies and is responsible for receptor binding, host range, hemagglutination, pathogenicity, and virulence of the virus (5–9). In the 98 CPV VP2 sequences obtained in this study, a total of 15 non synonymous changes (G4E, A5G, P13S, M87L, I101T, F267Y, S297A, A300G, D305Y, Y324I, Q370R, N375D, N426D/E, T440A, and I447M) were observed (Table 1). Of the above VP2 amino acid mutations, two altered amino acid residues that fell both within the footprint of the transferrin receptor (TfR) and the defined monoclonal antibody recognition region (VP2 positions 87 and 300); amino acids 297, 305, 426, and 440 were located in the antibody recognition region (48). A previous study indicated that the major neutralizing epitopes of CPV were located at the VP2 amino terminus, and there were three neutralizing epitopes within the first 37 amino-terminal residues of the VP2 protein (49). The VP2 G4E, A5G, and P13S mutations detected in this study were all located in the core regions of the neutralizing epitope. Given the mutations of amino acid residues fell within the TfR binding region or antibody recognition regions in the VP2 protein, it is speculated that the conventional commercial vaccines prepared with the original CPV-2 genotype might provide insufficient protection against the prevalent CPV-2c strain. In addition, the prevalent CPV-2c can infect dogs immunized with commercial vaccines and cause serious clinical manifestations, which further support this hypothesis (see Table S1).

The majority of the CPV-2c strains (94.4%, 85/90) in our study carried an amino acid mutation in VP2 (i.e., A5G), which was first reported in 2015 (21). The recent common ancestor time analysis results indicated that the A5G mutation may have appeared around November 2011. Following a statistical analysis of the Chinese strains that appeared in A5G, it found that the mutation first appeared in the strains isolated in 2013, and it has recently increased with each year (see Fig. S2). The A5G mutation often appears together with F267Y, Y324I, and Q370R mutations in Chinese recently popular CPV-2c strains (42, 50). Positive mutation sites and a parallel evolution analysis found that amino acid position 324 in the VP2 protein belonged to positive selection sites, one amino acid mutation F267Y belonged to a parallel mutation, and aa positions 5 and 370 of VP2 were under both positive selection and parallel evolution. In addition, the VP2 proteins of all CPV-2c strains (*n* = 85) containing the mutation of A5G in this study carried the F267Y, Y324I, and Q370R mutations. It is speculated that the emergence of the A5G, F267Y, Y324I, and Q370R mutations might have had a positive effect on the ability of CPV-2c to infect dogs, which might represent the reason for the phenomenon observed in the MCC tree that the CPV-2c variants had a greater capacity to infect dogs.

VP1 contains the complete VP2 sequence, as well as a 143-residue unique N-terminal sequence. Therefore, the aa changes (G147E, A148G, P156S, M230L, I244T, F410Y, S440A, A443G, D448Y, Y467I, Q513R, N518D, N569E, T583A, and I590M) of the VP1 protein were lying in the corresponding encoded VP2 protein (G4E, A5G, P13S, M87L, I101T, F267Y, S297A, A300G, D305Y, Y324I, Q370R, N375D, N426E, T440A, and I447M). In addition, the VP1 mutations in this study were primarily concentrated in aa residue 131 (A131T and A131N) and were located in a T cell epitope (peptide 119PKIFINLAKKKKAG132) of the V1 protein (51). However, we need to further confirm whether these mutations will affect the function of the T cell epitope.

Currently, limited studies are available on the CPV non-structural genes (44). I60V, Y544F, E545V, and L630P are the most frequently mutations in the NS1 gene of Asian-origin CPV-2c variants (52), which were identified in numerous CPV-2c strains (*n* = 79) in this study. Apart from the mutation points above, another 8 aa mutations (K19R, N356S, E572K, E583K, P590R, V596A, P599S, and N624K) were identified in the NS1 protein. The mutation NS1 P590R occurred in three CPV-2c strains here, but it was reported only once in a Chinese CPV-2c strain (GenBank no. MH476587; collection date, 2017). According to established research models (12), the K19R and I60V mutations were located in the N-terminal origin of the replication domain, the N356S mutation was located in the SF3 helicase domain, and the N624K and L630P mutations were located in the C-terminal transactivation domain. Thus, these mutations may have an impact on the function of the domains in which they are located. The other NS1 mutations (Y544F, E545V, E572K, E583K, P590R, V596A, and P599S) were located between the SF3 helicase domain and the C-terminal activation domain of NS1. The biological role of these amino acid mutations requires further exploration.

Notably, five CPV-2c mutants (HN-019, HN-051, HN-056, HN-057, and HN-058) in this study harbored the VP2 I447M mutation. There was an uncommon N115D mutation in the NS2 protein of the HN-019, HN-056, HN-057, and HN-058 mutants. Currently, the CPV-2c variant carrying the VP2 I447M mutation mainly appeared in Vietnam and has been present in Vietnamese dogs since about 2017 (53, 54), and the NS2 N115D mutation was only found in one Vietnamese CPV-2c strain (GenBank no. MT106238; collection date, 2017). Further analysis revealed that the HN-051 strain (VP2 A5G, and I447M) was highly homologous to the Vietnamese reference strain HCM01 (MT106234) with 100.0 and 99.9% amino acid homologies in VP2 and NS1, respectively. The phylogenetic results of VP2 showed that HN-051 was clustered in a small clade with the Vietnamese reference strains HCM01 (MT106234), HCM03 (MT106237), and HN08 (MT106230). Although the HN019, HN056, HN057, and HN058 strains were clustered in a large clade with the Vietnamese reference strain described above, they clustered individually to form a small independent clade. A phylogenetic analysis based on NS1 showed that the HN051 strain formed a large cluster with most CPV-2c strains in this study and the Vietnamese reference strains HCM01 (MT106234), HCM03 (MT106237), and HN08 (MT106230). However, the HN019, HN056, HN057, and HN058 strains were clustered in a clade with some new CPV-2a reference strains. Therefore, we boldly speculate that the HN051 strains with the VP2 I447M mutation may be imported from abroad, and the HN019, HN056, HN057, and HN058 strains with the VP2 I447M mutation may have been derived from parallel mutations of local strains. This is also the first report that the CPV-2c (NS2 115D, VP2 5A 447M) and CPV-2c (NS2 115N, VP2 5G 447M) mutants existed in China. In addition, the parallel evolution analyses showed that I447M belongs to parallel evolution; however, the biological significance of the mutation at this site remains unknown.

To further explore the potential recombination events of CPV-2 in this study, we performed a recombination analysis of all available CPV-2 genomes using the Recombination Detection Program, v.4.43 (RDP4). As a result, no significant (4 of 7 RDP methods with a *P* value of <10^−6^) recombination events were determined in any CPV-2 strains (data not shown).

In conclusion, a large-scale epidemiological survey found that CPV-2c has become the dominant genotype in Henan province and includes several different mutant genotypes which differed from CPV-2c discovered earlier in China. Based on the VP2 protein of the Chinese CPV-2 strains available in GenBank database and this study, an epidemiological survey indicated that CPV-2c has replaced new CPV-2a as the dominant genotype in China since about 2018. The maximum clade credibility (MCC) time-resolved tree, positive selection, and parallel evolution analyses of CPV-2 in China suggest that the emergence of the A5G, F267Y, Y324I, and Q370R mutations in CPV-2c may have a positive effect on the infectivity and transmissibility to dogs. Moreover, the CPV-2c variants with Vietnamese CPV-2c mutation characteristics (VP2 I447M) were reported for the first time in China. Thus, this study provides valuable information regarding the evolution of CPV-2 strains in China.

## MATERIALS AND METHODS

### Sample collection.

A total of 111 fecal samples or rectal swabs from dogs with clinical signs of gastroenteritis (vomiting and/or diarrhea) were collected from animal hospitals located in 10 cities (Zhengzhou, Pingdingshan, Luoyang, Anyang, Xinxiang, Sanmenxia, Xinyang, Jiaozuo, Luohe, and ZhouKou) of Henan province in China from June 2020 to January 2021. The fecal and rectal swab samples were stored in 1.5 mL of sterile phosphate-buffered saline (pH 7.2). The samples were either used immediately for DNA extraction or stored at −80°C until use. Details of the samples are summarized in Table S1.

### Detection of CPV-2 and near full-length genome sequencing.

DNA was extracted from the clinical samples using a TIANamp Virus DNA/RNA kit (Tiangen, Beijing, China) in accordance with the manufacturer’s instructions and subsequently screened the presence of the CPV-2 genome by PCR using specific primers VP2-F1/R1 (see Table S2 in the supplemental material) as described previously (26). In addition, all of the samples in this study were also detected for the presence of canine rotavirus, canine coronavirus (CoV), and canine adenovirus (CAV) in dogs using PCR or RT-PCR as described previously (55–57). Near full-length genome was amplified by three independent overlapping PCR assays using specific primers NS1-F/R (58), VP2-F2/R2, and NS1-VP2-F/R (see Table S2). The sequences were assembled and analyzed with DNAMAN and Lasergene.v7.1 and then submitted to the GenBank databases.

### Data collection and cleaning.

Among all of the Chinese CPV-2 VP2 sequences collected from GenBank (accessed on 25 June 2021), low-quality sequences and ORFs with <90% integrity were removed by using a previously described method (59). The sequences were aligned using MAFFT (v7.453) (60). After removing highly similar sequences with a 99.9% threshold by BioAider (V1.334) (61), 724 high-quality CPV VP2 sequences with metadata (collection date, host, and location) from China were obtained by pooling our sequences (*n* = 98).

### Phylogenetic analyses.

Neighbor-joining trees were constructed using a p-distance model and bootstrapping at 1,000 replicates. To estimate the time signal of collected sequences, a root-to-tip distance analysis was evaluated by Timetree (v0.8.1) (62). BEAST (v1.10.4) (63) was executed with the GTR+G4 substitution model and Bayesian Skygrid coalescent (BSG) model under the uncorrelated relaxed clock using Markov chain Monte Carlo (MCMC). The MCMC chain length was 200 million generations and sampled every 20,000 steps. Logfile was inspected by the Tracer (v1.7.1) to guarantee all effective sample sizes greater than 200 (ESS > 200). Finally, an MCC tree was generated using TreeAnnotator (part of BEAST v1.10.4). The ggtree (64) and ggplot2 (65) packages in R (v4.0.5) were used to visualize the tree. To investigate the demographic history of the CPV-2 in China, the effective population dynamic across time was reconstructed using Tracer.

### Amino acid mutations, adaptation, and parallel evolution.

Sequences were edited and aligned with those of reference strains retrieved from the GenBank database (http://www.ncbi.nlm.nih.gov) using AliView (v1.28). The NS1 or VP2 amino acid sequences were deduced from their ORFs. The option to toggle conserved sites with a conservation score of 100% was employed to highlight outlier amino acids (66).

To determine which amino acids in the VP2 coding region were under positive selection, Hyphy (v2.5.2) was used to estimate the ratio of nonsynonymous to synonymous substitution rates (67–69). The mixed-effects model of the evolution method (MEME) was used to estimate the selection pressure of the mutated amino acid sites. Treesub programs (https://github.com/tamuri/treesub, last accessed on 14 December 2021) were utilized to infer amino acid substitutions along the branches. ProtParCon (70) was used to evaluate the sites that underwent parallel and convergent evolution among the internal node by reconstructing ancestral nodes to the tips based on sequences.

### Data availability.

The CPV-2 sequences obtained in our study are available from the National Center for Biotechnology Information (GenBank accession numbers ON322751 to ON322848).
