# Supplementary material for: Prevalence and Characteristics of Canine Parvovirus Type 2 in Henan Province, China
Source: Microbiol Spectr. 2022 Nov 15;10(6):e01856-22. doi: 10.1128/spectrum.01856-22 (PMC9769957; doi:10.1128/spectrum.01856-22)
Supplement: Supplemental file 1 — Fig. S1 and S2 and Tables S1 to S3. Download spectrum.01856-22-s0001.pdf, PDF file, 0.4 MB [file spectrum.01856-22-s0001.pdf]

Supplementary Information

for

Prevalence and characteristics of canine parvovirus type 2 in Henan province, China

Pengfei Fu, Dongchang He, Xuan Cheng, Xinrui Niu, Congrong Wang, Yiqian Fu, Kun Li, Heshui Zhu, Weifei Lu, Jiang Wang, Beibei Chu

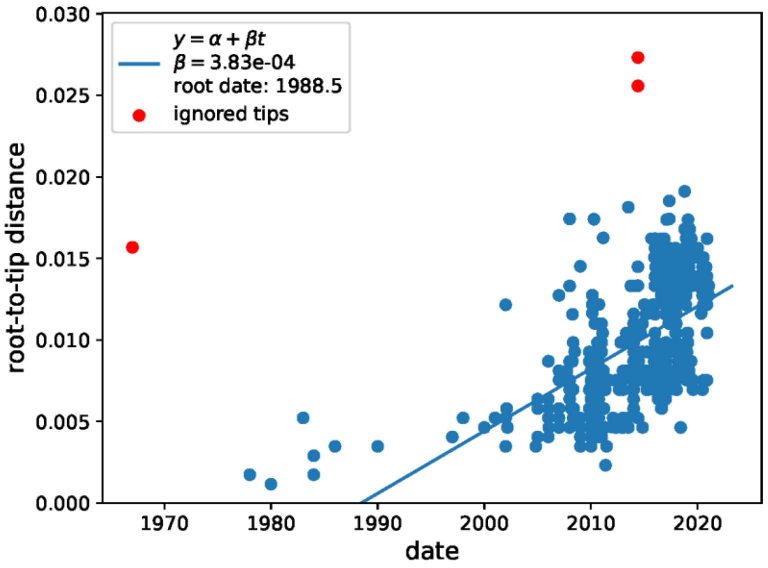

Figure S1. Temporal signal evaluation of CPV-2 VP2 gene.

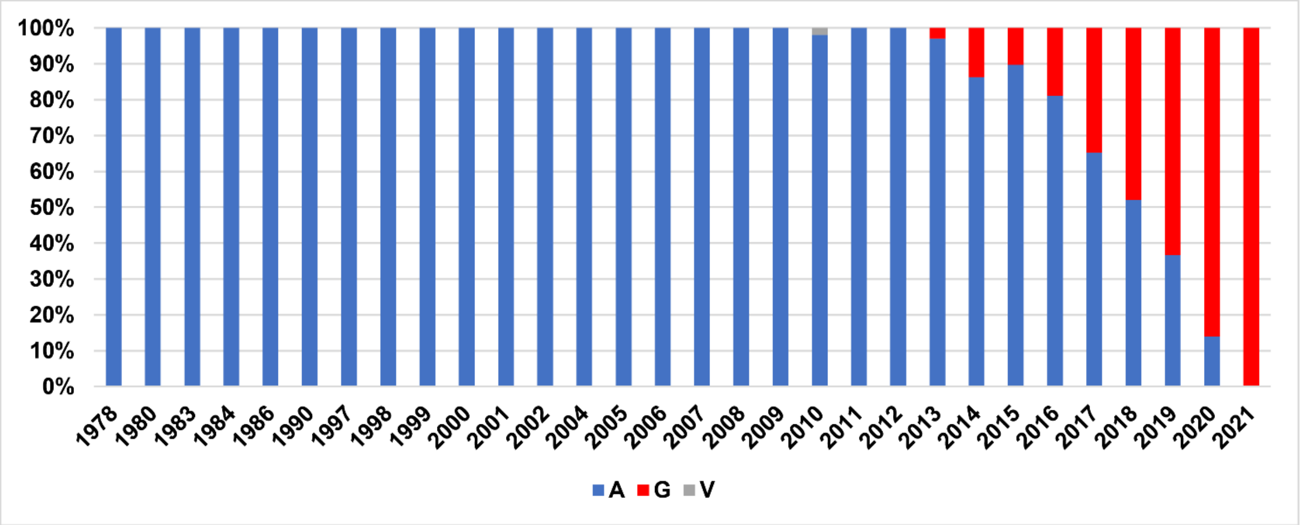

Figure S2. The proportion of VP2 A5G in China.

Supplementary Table 1 Clinical data and genotype of the CPV-2 positive samples in this study

Strain	Location	Sampling time[†]	Breed	Age (month)[‡]	Sex[§]	Vaccination	Genotype	CoV	CAV-Ⅰ/CAV-Ⅱ	CRV	Sample type
HN-002	Zhengzhou	2020/June	Husky	4	M	+	CPV-2c	-	-/-	-	rectal swabs
HN-003	Zhengzhou	2020/June	Chinese Rural Dog	2	M	+	new CPV-2a	-	+/+	-	rectal swabs
HN-004	Zhengzhou	2020/June	Chinese Rural Dog	5	M	+	CPV-2c	-	-/-	-	rectal swabs
HN-005	Zhengzhou	2020/June	Labrador	3	F	+	CPV-2c	-	-/-	-	feces
HN-006	Zhengzhou	2020/June	Pug	20	M	-	CPV-2c	-	-/-	-	feces
HN-007	Zhengzhou	2020/June	Teddy	2	F	+	CPV-2c	-	-/-	-	feces
HN-008	Zhengzhou	2020/June	Italian Greyhound	3	M	unknown	CPV-2c	-	-/-	-	feces
HN-009	Pingdingshan	2020/July	Teddy	6	F	-	CPV-2c	-	-/-	-	feces
HN-010	Pingdingshan	2020/July	Bichon	2	F	-	CPV-2c	-	-/-	-	feces
HN-011	Zhengzhou	2020/July	Yorkshire Terrier	2	F	-	CPV-2c	-	-/-	-	rectal swabs
HN-012	Zhengzhou	2020/Aug	Pug	2	F	-	CPV-2c	-	-/-	-	rectal swabs
HN-013	Luoyang	2020/Aug	Golden Retriever	4	M	unknown	new CPV-2a	+	-/-	-	rectal swabs
HN-014	Luoyang	2020/Aug	Husky	4	M	unknown	new CPV-2a	+	-/+	-	rectal swabs
HN-015	Luoyang	2020/Aug	Poodle	3	F	unknown	new CPV-2a	+	-/+	-	rectal swabs
HN-016	Luoyang	2020/Aug	Pekingese	3	F	unknown	new CPV-2a	+	-/+	-	rectal swabs
HN-017	Anyang	2020/July	Golden Retriever	2	M	-	CPV-2c	+	-/-	-	rectal swabs
HN-018	Anyang	2020/Aug	Golden Retriever	2	M	-	CPV-2c	-	-/-	-	rectal swabs
HN-019	Anyang	2020/Aug	Labrador	2	M	-	CPV-2c	-	-/-	-	rectal swabs
HN-021	Luoyang	2020/Aug	Pug	3	F	unknown	CPV-2c	-	-/-	-	rectal swabs
HN-022	Luoyang	2020/Aug	Chihuahua	3	F	unknown	CPV-2c	-	-/-	-	rectal swabs
HN-023	Luoyang	2020/Aug	Golden Retriever	5	F	unknown	CPV-2c	-	-/-	-	rectal swabs
HN-024	Luoyang	2020/Aug	Border Collie	5	M	unknown	CPV-2c	-	-/-	-	rectal swabs
HN-025	Luoyang	2020/Aug	Chow Chow	4	M	unknown	CPV-2c	-	-/-	-	rectal swabs
HN-026	Xinxiang	2020/July	Husky	2	M	-	CPV-2c	-	-/-	-	feces
HN-027	Xinxiang	2020/July	Samoyed	2	M	-	new CPV-2a	-	-/-	-	feces
HN-028	Xinxiang	2020/Aug	Pomeranian	3	M	-	CPV-2c	+	-/-	-	feces
HN-029	Luoyang	2020/Aug	Poodle	4	M	unknown	CPV-2c	-	-/-	-	rectal swabs
HN-030	Luoyang	2020/Aug	Pug	6	F	unknown	CPV-2c	-	-/-	-	rectal swabs
HN-031	Luoyang	2020/Aug	Pug	5	M	unknown	CPV-2c	-	-/-	-	rectal swabs
HN-033	Luoyang	2020/Aug	Pomeranian	3	M	unknown	CPV-2c	-	-/-	-	feces
HN-034	Luoyang	2020/Aug	Yorkshire Terrier	2	F	unknown	CPV-2c	-	-/-	-	feces
HN-035	Sanmenxia	2020/July	German shepherd	3	M	unknown	CPV-2c	-	-/-	-	rectal swabs
HN-036	Sanmenxia	2020/Aug	Alaskan	2	F	unknown	new CPV-2a	+	-/-	-	rectal swabs
HN-038	Zhengzhou	2020/Aug	Golden Retriever	6	M	unknown	CPV-2c	-	-/-	-	rectal swabs
HN-039	Zhengzhou	2020/Aug	Samoyed	2	F	-	CPV-2c	-	-/-	-	rectal swabs
HN-040	Pingdingshan	2020/Aug	Shiba Inu	2	F	-	CPV-2c	-	-/-	-	feces
HN-041	Xinyang	2020/Aug	Crossbred Dog	1	F	-	CPV-2c	-	-/-	-	feces
HN-042	Xinyang	2020/Aug	Labrador	2	F	+	CPV-2c	-	-/-	-	feces
HN-043	Jiaozuo	2020/Aug	Chihuahua	2	F	unknown	CPV-2c	-	-/-	-	rectal swabs
HN-044	Jiaozuo	2020/Aug	Pekingese	5	F	unknown	CPV-2c	-	-/+	-	rectal swabs
HN-045	Anyang	2020/Aug	Golden Retriever	2	M	unknown	CPV-2c	-	-/+	-	rectal swabs
HN-046	Zhengzhou	2020/Aug	Belgian Malinois	2	F	unknown	CPV-2c	+	-/-	-	rectal swabs
HN-047	Anyang	2020/Aug	Bichon frise	1	M	unknown	CPV-2c	-	-/-	-	rectal swabs
HN-048	Anyang	2020/Aug	Golden Retriever	2	M	unknown	CPV-2c	-	-/-	-	rectal swabs
HN-049	Anyang	2020/Sep	Corgi	2	M	unknown	CPV-2c	+	-/-	-	rectal swabs
HN-050	Anyang	2020/Aug	Golden Retriever	2	M	unknown	CPV-2c	-	-/-	-	rectal swabs
HN-051	Pingdingshan	2020/Sep	Pomeranian	3	F	unknown	CPV-2c	-	-/-	-	rectal swabs
HN-055	Pingdingshan	2020/Sep	Chow Chow	3	M	unknown	CPV-2c	-	-/-	-	rectal swabs
HN-056	Anyang	2020/Oct	Schnauzer	4	M	unknown	CPV-2c	-	-/-	-	rectal swabs
HN-057	Anyang	2020/Oct	Poodle	6	F	unknown	CPV-2c	-	-/+	-	rectal swabs
HN-058	Anyang	2020/Oct	Golden Retriever	6	M	unknown	CPV-2c	-	-/+	-	rectal swabs
HN-059	Anyang	2020/Oct	Bichon frise	4	M	unknown	CPV-2c	-	-/-	-	rectal swabs
HN-060	Anyang	2020/Oct	Golden Retriever	2	M	unknown	CPV-2c	-	-/+	-	rectal swabs
HN-061	Anyang	2020/Oct	Italian Greyhound	3	M	unknown	CPV-2c	-	-/-	-	rectal swabs
HN-062	Anyang	2020/Oct	Belgian Malinois	5	M	unknown	CPV-2c	+	-/-	-	rectal swabs
HN-063	Anyang	2020/Oct	Pomeranian	2	F	unknown	CPV-2c	-	-/-	-	rectal swabs
HN-064	Anyang	2020/Oct	Samoyed	3	M	unknown	CPV-2c	+	-/-	-	rectal swabs
HN-065	Anyang	2020/Oct	Corgi	6	F	unknown	CPV-2c	-	-/-	-	rectal swabs
HN-066	Anyang	2020/Oct	Samoyed	4	M	unknown	CPV-2c	-	-/-	-	rectal swabs
HN-068	Xinxiang	2020/Aug	Schnauzer	3	M	+	CPV-2c	-	-/-	-	feces
HN-069	Xinxiang	2020/Sep	Poodle	3	M	+	CPV-2c	-	-/-	-	feces
HN-070	Xinxiang	2020/Aug	Pomeranian	2	F	-	CPV-2c	-	-/-	-	feces
HN-071	Xinxiang	2020/Sep	Poodle	3	F	+	CPV-2c	-	-/-	-	feces
HN-072	Xinxiang	2020/Sep	Chinese Rural Dog	3	M	+	CPV-2c	-	-/-	-	feces
HN-073	Xinxiang	2020/Sep	Crossbred Dog	3	M	-	CPV-2c	-	-/-	-	feces
HN-074	Xinxiang	2020/Sep	Corgi	5	M	+	CPV-2c	-	-/-	-	feces
HN-075	Xinxiang	2020/Sep	Schnauzer	2	F	-	CPV-2c	-	-/-	-	feces
HN-076	Xinxiang	2020/Sep	Chinese Rural Dog	6	F	-	CPV-2c	-	-/-	-	feces
HN-077	Xinxiang	2020/Oct	Chinese Rural Dog	4	M	-	CPV-2c	-	-/-	-	feces
HN-078	Xinxiang	2020/Aug	Samoyed	4	M	-	CPV-2c	-	-/-	-	feces
HN-079	Zhengzhou	2020/Nov	Corgi	2	M	unknown	CPV-2c	-	-/-	-	rectal swabs
HN-080	Zhengzhou	2020/Nov	Pomeranian	2	F	unknown	CPV-2c	-	-/-	-	rectal swabs
HN-081	Luohe	2020/Nov	Chihuahua	5	F	-	CPV-2c	-	-/-	-	rectal swabs
HN-082	Luohe	2020/Nov	Golden Retriever	4	F	-	CPV-2c	-	-/-	-	rectal swabs
HN-084	Luohe	2020/Nov	Pug	6	M	-	CPV-2c	-	-/-	-	rectal swabs
HN-085	Luohe	2020/Nov	Golden Retriever	7	F	-	CPV-2c	-	-/-	-	rectal swabs
HN-086	Luohe	2020/Nov	Samoyed	5	M	+	CPV-2c	+	-/-	-	rectal swabs
HN-087	Pingdingshan	2020/Nov	Bichon frise	5	M	-	CPV-2c	-	-/-	-	rectal swabs
HN-088	Pingdingshan	2020/Nov	Golden Retriever	3	M	-	CPV-2c	+	-/-	-	rectal swabs
HN-089	Pingdingshan	2020/Nov	Golden Retriever	3	F	-	new CPV-2a	-	-/-	-	rectal swabs
HN-090	Pingdingshan	2020/Nov	Chihuahua	2	M	-	CPV-2c	-	-/-	-	rectal swabs
HN-091	Pingdingshan	2020/Nov	Poodle	3	F	-	CPV-2c	-	-/-	-	rectal swabs
HN-092	Pingdingshan	2020/Nov	Teddy	3	F	-	CPV-2c	-	-/-	-	rectal swabs
HN-093	Pingdingshan	2020/Nov	Corgi	4	F	-	CPV-2c	-	-/-	-	rectal swabs
HN-097	ZhouKou	2020/Oct	Pomeranian	2	M	unknown	CPV-2c	-	-/-	-	rectal swabs
HN-098	ZhouKou	2020/Nov	Teddy	2	M	unknown	CPV-2c	-	-/-	-	rectal swabs
HN-099	ZhouKou	2021/Jan	Labrador	6	F	unknown	CPV-2c	-	-/-	-	rectal swabs
HN-100	ZhouKou	2021/Jan	Pomeranian	3	M	unknown	CPV-2c	+	-/-	-	rectal swabs
HN-102	ZhouKou	2021/Jan	Teddy	4	M	unknown	CPV-2c	-	-/-	-	rectal swabs
HN-103	ZhouKou	2021/Jan	Poodle	7	M	unknown	CPV-2c	-	-/-	-	rectal swabs
HN-104	ZhouKou	2021/Jan	Golden Retriever	7	F	unknown	CPV-2c	-	-/-	-	rectal swabs
HN-105	ZhouKou	2021/Jan	Teddy	5	F	unknown	CPV-2c	+	-/-	-	rectal swabs
HN-106	ZhouKou	2021/Jan	Chihuahua	3	M	unknown	CPV-2c	-	-/-	-	rectal swabs
HN-107	ZhouKou	2021/Jan	Chihuahua	3	M	unknown	CPV-2c	-	-/-	-	rectal swabs
HN-108	ZhouKou	2021/Jan	Poodle	2	F	unknown	CPV-2c	-	-/-	-	rectal swabs
HN-109	ZhouKou	2021/Jan	Golden Retriever	3	M	unknown	CPV-2c	-	-/-	-	rectal swabs
HN-110	ZhouKou	2020/Aug	Bichon	2	M	unknown	CPV-2c	-	-/-	-	rectal swabs
HN-111	ZhouKou	2020/Nov	Alaska	12	M	unknown	CPV-2c	-	-/-	-	rectal swabs

Supplementary Table 2 The primers used in this study

Virus	Name of Primer	Sequence (5’-3’)	Expected product/bp	Applications	The origin of primers
CPV	VP2-F1	GTACATTTAAATATGCCAGA	452	To detect VP2 gene	Mochizuki et al., 1996
	VP2-R1	ATTAATGTTCTATCCCATTG			
	NS1-F	GACCGTTACTGACATTCGCTTC	2251	To amplify the complete gene of NS1	Mira et al., 2018
	NS1-R	GAAGGGTTAGTTGGTTCTCC			
	VP2-F2	CAATTGCTCCAGTATTAACTGATAC	2048	To amplify the complete gene of VP2	In this study
	VP2-R2	CTATTTCTTACAGTTATTGTATAC			
	NS1-VP2-F	TCCAGATACGCCTATTGC	873	To amplify the DNA sequence between NS1 and VP2	In this study
	NS1-VP2-R	CTGCTTGAGTTTGCTGTG			
CAV	HA1	CGCGCTGAACATTACTACCTTGTC	CAV-1:509/CAV-2:1031	To amplify the sequences of E3 and flanking regions	Hu et al., 2001
	HA2	CCTAGAGCACTTCGTGTCCGCTT			
CRV	VP6-F	GACGGVGCRACTACATGGT	379	To detect VP6 gene	Charoenkul et al., 2021
	VP6-R	GTCCAATTCATNCCTGGTGG			
CCV	CCV1	TCCAGATATGTAATGTTCGG	230	A nested PCR to detect M gene	Pratelli et al., 1999
	CCV2	TCTGTTGAGTAATCACCAGCT			
	CCV3	GGTGTCACTCTAACATTGCTT			

Supplementary Table 3 Result of positive selection sites for CPV VP2

Codon	Episodic selection detected?	Most common codon substitutions at this site
5	Yes, p = 0.0646	GCA>GGA
63	Yes, p = 0.0002	GCA>CGA
91	Yes, p = 0.0081	GCA>AGA, GCA>GTA, GCA>TCA
92	Yes, p = 0.0105	GTT>TTT
119	Yes, p = 0.0009	GTT>AAT
173	Yes, p = 0.0899	TTG>TTA
174	Yes, p = 0.0011	ATG>TGG
177	Yes, p = 0.0047	TTA>ACA, TTA>TCA
188	Yes, p = 0.0005	GCA>CAG, GCA>GCG
195	Yes, p = 0.0006	TTG>TTA
270	Yes, p = 0.0553	TGT>TGC
324	Yes, p = 0.0000	ATT>TAT
370	Yes, p = 0.0914	CGA>CAA
419	Yes, p = 0.0226	AAC>AGC, AAC>GAC
426	Yes, p = 0.0103	AAT>GAT
457	Yes, p = 0.0068	TTA>TAC, TTA>TCA, TTA>TTG
571	Yes, p = 0.0321	ATT>AAA, ATT>ATC

Found 17 sites under episodic diversifying positive selection at $p \leq 0.1$
